# Exploring Evolutionary Relationships within Neodermata Using Putative Orthologous Groups of Proteins, with Emphasis on Peptidases

**DOI:** 10.3390/tropicalmed8010059

**Published:** 2023-01-12

**Authors:** Víctor Caña-Bozada, Mark W. Robinson, David I. Hernández-Mena, Francisco N. Morales-Serna

**Affiliations:** 1 Centro de Investigación en Alimentación y Desarrollo, Mazatlán 82112, Mexico; 2 School of Biological Sciences, Queen’s University Belfast, 19 Chlorine Gardens, Belfast BT9 5DL, UK; 3 Centro de Investigación y de Estudios Avanzados, Instituto Politécnico Nacional, Unidad Mérida, Mérida 97310, Mexico; 4 Instituto de Ciencias del Mar y Limnología, Universidad Nacional Autónoma de México, Mazatlán 82040, Mexico

**Keywords:** Platyhelminthes, monogenea, trematoda, cestoda, phylogenomic, parasites

## Abstract

The phylogenetic relationships within Neodermata were examined based on putative orthologous groups of proteins (OGPs) from 11 species of Monogenea, Trematoda, and Cestoda. The dataset included OGPs from BUSCO and OMA. Additionally, peptidases were identified and evaluated as phylogenetic markers. Phylogenies were inferred using the maximum likelihood method. A network analysis and a hierarchical grouping analysis of the principal components (HCPC) of orthologous groups of peptidases were performed. The phylogenetic analyses showed the monopisthocotylean monogeneans as the sister-group of cestodes, and the polyopisthocotylean monogeneans as the sister-group of trematodes. However, the sister-group relationship between Monopisthocotylea and Cestoda was not statistically well supported. The network analysis and HCPC also showed a cluster formed by polyopisthocotyleans and trematodes. The present study supports the non-monophyly of Monogenea. An analysis of mutation rates indicated that secreted peptidases and inhibitors, and those with multiple copies, are under positive selection pressure, which could explain the expansion of some families such as C01, C19, I02, and S01. Whilst not definitive, our study presents another point of view in the discussion of the evolution of Neodermata, and we hope that our data drive further discussion and debate on this intriguing topic.

## 1. Introduction

The Neodermata are a group of Platyhelminthes that is made up of metazoan parasites of three classes: Monogenea (primarily ectoparasitic), Trematoda (endoparasitic flukes), and Cestoda (endoparasitic tapeworms). These parasites infect a variety of vertebrate hosts and can cause disease in humans, farmed fish, livestock, and domestic animals. Although the existence of the Neodermata as a monophyletic group is well established, the phylogenetic relationships between the neodermatan classes remain under debate [1]. The clarification of this is important for understanding the evolutionary origins of endo- and ectoparasitism as well as the complex life cycles of flatworms [1,2,3,4]. 

One of the major controversies is the monophyly of Monogenea [5,6]. This class is divided into two subclasses (Polyopisthocotylea and Monopisthocotylea), that form a well-supported monophyletic group [5,7]. However, some studies, based on morphological [8,9] and molecular characters [3,10], suggest that Polyopisthocotylea and Monopisthocotylea do not occupy the same clade.

One of the better supported hypotheses, based on molecular data, is the early divergence of Monogenea and the sister-group relationship of Trematoda and Cestoda [6]. Nonetheless, there is also some evidence that infers a closer relationship of Monogenea (especially Monopisthocotylea) with Cestoda [1,3,11]. Some of these discrepancies may be due to the variety and quality of sequence data used or because some taxa are poorly represented (e.g., Polyopisthocotylea). Moreover, platyhelminth mitochondrial genomes have a high substitution rate (around four times that of other bilaterian taxa) and so may not be ideal for this type of analysis [12,13]. Thus, analyses based on multiple nuclear genes may provide greater resolution of the phylogenetic relationships with Neodermata [12,14,15,16,17]. Currently, this task is facilitated by BUSCO and OMA, which are two leading programs for identifying putative orthologous groups of genes and proteins [18,19].

As an alternative to the use of complete genomes or transcriptomes, the use of a protein family with homology in a wide range of species could be considered in phylogenetic analyses [20]. In this context, given that neodermatan peptidases are important virulence factors that perform roles essential for parasitism, including feeding, migration, and avoidance/modulation of the host immune response [21,22,23,24,25], it would be interesting to explore their use as phylogenetic markers. Although characteristics such as lifestyle (free-living, ectoparasites, and endoparasites), host niche (gills, intestine, liver, etc.), and feeding (by degradation of host mucus, tissue, and blood or by direct uptake of digestion products) are variable among monogeneans, trematodes, and cestodes [3], secreted helminth peptidases (operating at the host-parasite interface) are under selective pressure from the host and thus may respond similarly and leave genuine phylogenetic signals across diverse lineages. For instance, positive selection can accelerate the fixation of advantageous mutations that enhance or refine the functions of the ancestral gene [26], as has been seen with the expansion of a family of cathepsins L with distinct but overlapping substrate specificities in the trematode, *Fasciola hepatica* [27]. Thus, the aim of this study was to explore the evolutionary relationships within Neodermata using putative orthologous groups of proteins (OGPs) retrieved from BUSCO and OMA, and to evaluate the use of peptidases as markers for phylogenetic studies.

## 2. Materials and Methods

### 2.1. Evolutionary Relationships of Neodermata

#### 2.1.1. Phylogenetic Analysis of BUSCO and OMA OGPs

Two phylogenetic analyses were performed using putative OGPs obtained from the genomes (g), transcriptomes (t), or EST sequences of 11 species of Neodermata: the monopisthocotyleans *Rhabdosynochus viridisi* (t), *Scutogyrus longicornis* (t), *Gyrodactylus salaris* (g), and *Neobenedenia melleni* (EST) [28,29]; the polyopisthocotyleans *Protopolystoma xenopodis* (g) and *Eudiplozoon nipponicum* (t) [29,30]; the cestodes *Echinococcus multilocularis* (g), *Hymenolepis microstoma* (g), and *Taenia asiatica* (g) [29]; and the trematodes *F. hepatica* (g) and *Schistosoma mansoni* (g) [29]. The free-living platyhelminths *Bothrioplana semperi* (g), belonging to the order Bothrioplanida, and *Schmidtea mediterranea* (g), belonging to the order Tricladida, were used as outgroups [12,29]. The first analysis included single-copy OGPs retrieved from BUSCO v4 [31], using the core metazoan dataset, which contains 978 genes and the script BUSCO_phylogenomics.py with parameters “-supermatrix” and “-psc 70”. The second analysis included simply OGPs (OMA Groups) obtained through OMA Standalone [32], using the script filter_groups.py [19]. OMA Groups contained a maximum of one representative gene per species. When multiple co-orthologs exist, OMA selected one to be in the OMA Group. Only the OGPs present in at least 10 species were included in the analyses.

The BUSCO proteins used in the phylogenetic analyses were annotated with the odb10 database [33], whereas the OMA proteins were annotated using BLASTp [34] against the UniProtKB/Swiss-Prot database (e-value < 1 × 10^−4^). In addition, the proteins were mapped to Gene Ontology (GO) terms using the PANNZER2 web server [35]. The visualisation and GO term enrichment analysis were performed in WEGO [36].

The OGPs were aligned with Muscle v3.8.31 [37], trimmed with trimAL [38] using the automated mode (-automated1), and concatenated. The best evolutionary models were obtained with the ModelFinder program [39]. Phylogenetic trees were constructed in IQ-TREE v1.6.12 [40], using the Shimodaira–Hasegawa-like approximate likelihood ratio test (SH-aLRT) (1000 replicates) and 1000 ultrafast bootstrap approximations to calculate the support values of the clades. Phylogenetic trees were also constructed in RAxML v8 [41], with 1000 bootstrap (Bs) iterations to calculate the support values of the clades. The trees were visualised with FigTree v1.4.2 (http://tree.bio.ed.ac.uk/software/figtree/, accessed on 1 November 2021). The trees were constructed using the models LG + F + R4 and LG + F + G + I for IQ-TREE and RAxML, respectively. Constrained trees were constructed in IQ-TREE (using the -g option), using alternative scenarios obtained from the literature as a guide (see Table 1) and the LG + F + R4 model. To determine whether the topology found in this study was significantly better than constrained trees, tree topology tests were performed in IQ-TREE using the LG + F + R4 evolutionary model with the options: -au, -zb.

#### 2.1.2. Phylogenetic Analysis of Peptidases

Peptidase and peptidase inhibitors were identified in the predicted proteins of the aforementioned 11 species of Neodermata and two free-living platyhelminth species. To this end, we followed the recommendations of Rawlings et al. [48]. The predicted proteins were aligned against the MEROPS v12.3 database [48] using BLASTp (e-value < 1 × 10^−4^). To avoid the overestimation of sequences, only the protein isoform with the best e-value was considered in the analysis. It was not possible to identify the isoforms in all the files, so CD-HIT v4.8.1 [49] with 90% sequence identity was run for all the species, following Ji et al. [50]. The retained proteins were aligned against the NCBI non-redundant protein database for detecting potential contaminant sequences from bacteria, viruses, fish, or tetrapods. The resulting sequences were classified according to the MEROPS database and included aspartic, cysteine, glutamic, metallo, asparagine, mixed, serine, threonine, unknown peptidases, and inhibitor classes. Classically secreted proteins were identified with SignalP v4.1 [51], using default settings for eukaryotes. In addition, SecretomeP [52] with default options for mammalian organisms and filtered by NN-scores larger than 0.9 was used to identify non-classically secreted proteins.

The peptidases and peptidase inhibitors were submitted to OMA Standalone to obtain orthologous groups (OGs) and to make comparisons between neodermatans. In order to improve the retrieval of these OGs, the phylogenetic relationships data (in Newick format) were also submitted to OMA.

A multilocus phylogenetic analysis of peptidases was performed using the OMA Groups. This analysis included the peptidases present in at least 10 of the 13 species of platyhelminths. Each group of peptidases was aligned with MAFFT, using the parameters “-maxiterate 1000 and -localpair”, trimmed with the parameter “-gappyout”, and posteriorly concatenated. The selection of the best evolutionary models, the construction, and the visualisation of the trees were performed as described above. The trees were constructed using the models LG + F + R4 and LG + F + G + I for IQ-TREE and RAxML, respectively.

#### 2.1.3. Network Analysis of Peptidase OGs

A network analysis was performed using the OGs matrix of Hierarchical Orthologous Groups obtained from OMA Standalone. The Gephi v0.9.2 software [53] was used to construct a directed network. The communities (internal subdivisions) of the network were obtained following the modularity optimization method, based on the number of peptidases in each OG, which can be applied to weighted and directed graphs [54]. Some resolution values were evaluated, and the least variable resolution was chosen. The final parameters used in the analysis were resolution of 1.0, edge weights, and randomization [54]. In a network, the communities (clusters or modules) can be defined as groups of vertices that have common properties and/or perform similar roles, and that occur when the number of edges inside the subdivisions is higher than the expected number of internal edges that the same subgraph would have in the null model [55]. The values of modularity can fall between −1 and 1 and measure the density of links inside communities as compared to links between communities [54]. Higher positive values are linked to high densities of links inside communities and lower links between communities (better clustering), negative values indicate the opposite, and a zero value expresses link randoms in the cluster of networks [56]. The OGs associated with the formation of each cluster were retrieved. 

#### 2.1.4. Hierarchical Grouping Analysis in Principal Components of OGs

This analysis was performed with the R package “FactoMineR” [57] using the OGs matrix of Hierarchical Orthologous Groups. To prevent variables with large values from becoming dominant, the data were standardised with the option “scale.unit = TRUE”, which scaled the values to unit variance. Thereafter, the data were subjected to principal component analysis (PCA) to reduce the dimension of the data (principal components). Finally, the hierarchical clustering, using Euclidean distance matrices and Ward’s hierarchical clustering algorithm, was performed to obtain the clusters. Ward’s hierarchical clustering algorithm uses an ANOVA approach for calculating distances between clusters [58]. The hierarchical clustering analysis was performed using the parameter “nb.clust = 4” to obtain the same number of clusters observed in the phylogenomic analysis. The genes associated with each cluster were retrieved (*p*-value < 0.05) and compared with those obtained from the network analysis to obtain the main subfamilies of peptidases influencing the formation of the cluster. The information about the main subfamilies was visualised in a circus plot, using the R package “circlize” [59].

### 2.2. Mutation Rates in Secreted and Non-Secreted Peptidases

The paired OGs obtained from OMA Standalone (PairwiseOrthologs output in OMA) were analysed to estimate the rate of synonymous (Ks) and non-synonymous (Ka) substitutions among species of each class of platyhelminths. Briefly, each OG among species of the same class was aligned using MAFFT v7.31 [60] (-auto). The amino acid alignment was used as a guide to generate the nucleotide sequence alignment using ParaAT [61]. Then, the alignments were used to calculate Ks and Ka for each OG with the software Codeml (CodonFreq = 2, runmodel = −2) of the PAML package v4.8 [62]. The Ks and Ka were obtained using the pipeline PANAS [63]. Because the peptidases present high variability of nucleotides, all Ks values were retained, in contradiction with other analyses [64]. The Kruskal–Wallis test was used to evaluate significant differences (*p*-value < 0.05) of Ks and Ka between (1) single-copy OGs vs. multiple-copy OGs; (2) single-copy OGs secreted proteins vs. single-copy OGs non-secreted proteins; and (3) multiple-copy OGs secreted proteins and multiple-copy OGs non-secreted proteins.

### 2.3. Classification of the C01A and S01C Peptidase Subfamilies

The sequences of the C01A and S01C peptidase subfamilies were submitted to MOTIF Search (https://www.genome.jp/tools/motif/MOTIF.html, accessed on 1 December 2021) to retrieve the region of the Pfam domain Peptidase_C1 (PF00112) for the subfamily C01A, and Trypsin (PF00089) for the subfamily S01C. The members of these subfamilies were classified through phylogenetic analyses. Trees were constructed as described above, using only the approximate likelihood ratio test (1000 replicates), and visualised and annotated with FigTree and the ITOL web server v5 [65]. Bootstrap values above 80% were considered as significant. The phylogenetic analysis was performed using reference proteins of platyhelminths from the MEROPS database. In addition, the alignment of the sequences was used to identify the S2 subsite residues belonging to cathepsins L (positions 67, 157, and 205; papain numbering) in the subfamily C01A [27,66].

## 3. Results

### 3.1. Evolutionary Relationships of Neodermata

#### 3.1.1. Phylogenetic Analyses of BUSCO and OMA OGPs

**Figure 1:**
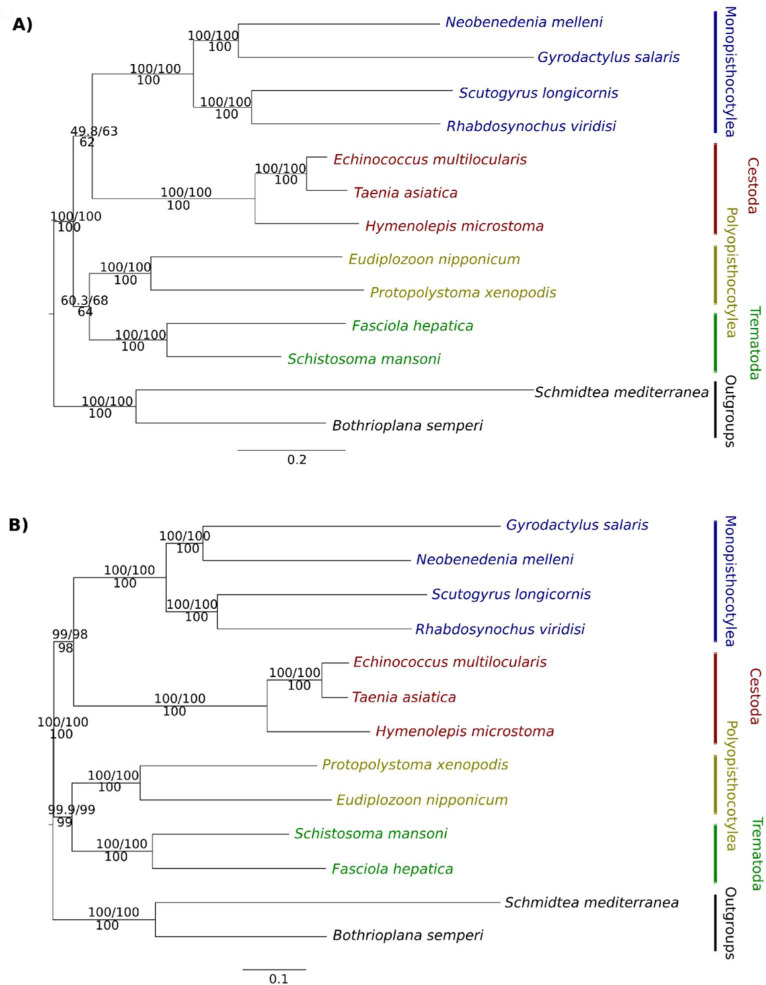
Phylogenetic trees of 13 platyhelminth species, based on (**A**) single-copy BUSCO OGPs and (**B**) simply OMA OGPs. Values above represent SH-aLRT support (%)/ultrafast bootstrap support (%) and below represent Maximum Likelihood rapid bootstrap support. The free-living platyhelminths *Bothrioplana semperi*, belonging to the order Bothrioplanida, and *Schmidtea mediterranea*, belonging to the order Tricladida, were used as outgroups.

The phylogenetic analyses were performed using 137 BUSCO OGPs (Supplementary File S1 and Table S1) and 479 OMA OGPs (Supplementary File S2 and Table S2). In the phylogenetic analysis using BUSCO OGPs, Monopisthocotylea appeared as the sister-group of Cestoda (Bs = 62%), whereas Polyopisthocotylea appeared as the sister-group of Trematoda (Bs = 64%). In turn, each of the classes and subclasses formed well-supported monophyletic groups (Bs = 100%) (Figure 1A). The tree constructed using OMA OGPs showed the same topology, with high level support in all the nodes (Bs > 97%, Figure 1B). The tree topology test revealed that by using BUSCO OGPs no scenario was rejected, whereas by using OMA OGPs only scenario 1 was not rejected (*p*-AU > 0.05) (Table 1). Constrained trees are shown in Supplementary Figure S1. The high Bs values observed in the tree based on OMA OGPs are possibly due to the use of a large dataset, because the same analysis with less data (200 OGPs) grouped Monopisthocotylea and Cestoda with support of 59%, and Polyopisthocotylea and Trematoda with 84% (tree not shown).

Details of the annotation of BUSCO and OMA OGPs is shown in Supplementary Tables S1 and S2. 

#### 3.1.2. Phylogenetic Analysis of Peptidases

**Figure 2:**
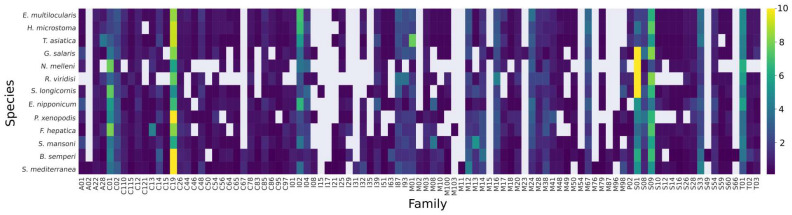
Heat map showing the proportion of peptidase and inhibitor families in each platyhelminth species.

A total of 3960 peptidases were identified in the 13 species of platyhelminths, distributed in 80 families across the five major classes (Supplementary Tables S3 and S4, Figures S2A and S3 and File S3). Serine peptidases were the most abundant class in monopisthocotyleans, whereas cysteine peptidases were the most abundant in polyopisthocotyleans, cestodes, trematodes, and free-living platyhelminths, except for *S. mansoni* and *T. asiatica*, where metallo peptidases were the most abundant. The most abundant peptidase families in all the platyhelminths studied were C19 (ubiquitinyl hydrolases), S01 (chymotrypsin family), S09 (prolyl oligopeptidases), C01 (papain family), and T01 (proteasome family) (Figure 2 and Supplementary Table S5). A total of 667 peptidase inhibitors belonging to 17 families were identified in all the platyhelminths (Supplementary Tables S3 and S5 and Figure S2B), with the family I02 (Kunitz) being the most abundant. Details of the identified peptidases are presented in Supplementary Results.

**Figure 3:**
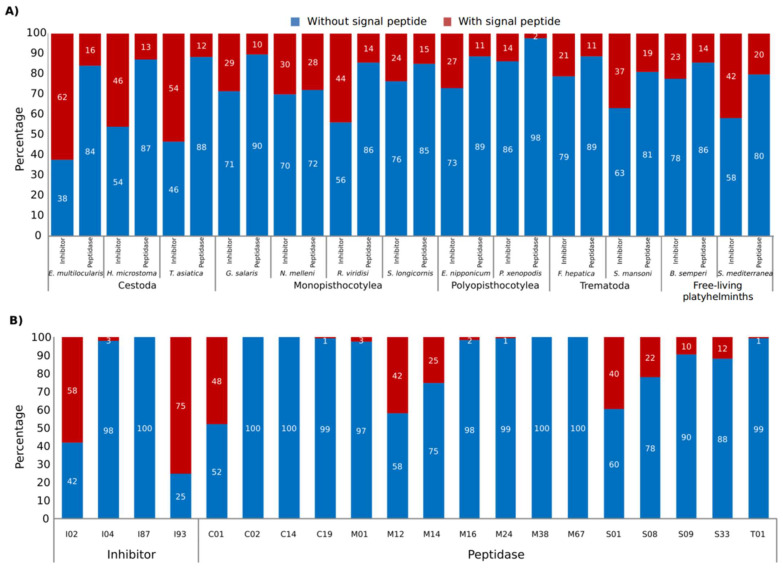
Peptidases and inhibitors with predicted signal peptides in the platyhelminth species analysed. (**A**) The proportion of peptidases and inhibitors with/without predicted signal peptides for secretion. (**B**) Proportion of secreted protein in the top 20 most abundant peptidase and inhibitor families.

SignalP predicted 565 peptidases (14.27%) and 237 peptidase inhibitors (35.53%) as classically secreted proteins (Figure 3A and Supplementary Table S6). The I02, I93, C01, M12, and S01 families presented a high proportion of proteins with signal peptides in comparison with other families (>40% of proteins with predicted N-terminal signal peptides) (Figure 3B). In addition, 79 peptidases (2%) and 18 peptidase inhibitors (2.7%) were predicted as non-classically secreted proteins, i.e., proteins lacking a signal peptide for secretion via the ER/Golgi pathway that exit the cell via atypical means [52].

**Figure 4:**
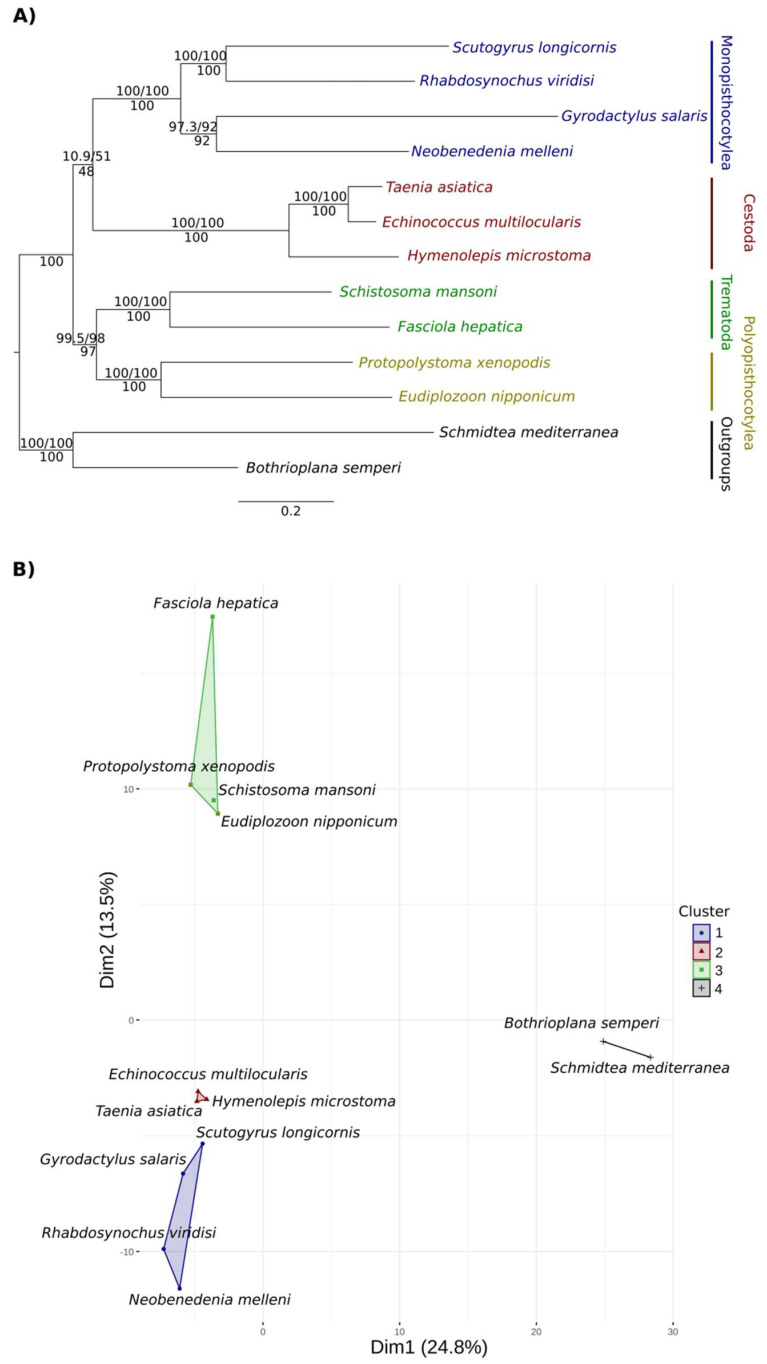
Peptidases and inhibitors of 13 platyhelminth species analysed. (**A**) Phylogenetic tree with values above representing SH-aLRT support (%)/ultrafast bootstrap support (%) and below representing Maximum Likelihood rapid bootstrap support. The free-living platyhelminths *Bothrioplana semperi*, belonging to the order Bothrioplanida, and *Schmidtea mediterranea*, belonging to the order Tricladida, were used as outgroups. (**B**) Formation of clusters according to their peptidases and inhibitors OGs using hierarchical grouping analysis in principal components.

A total of 531 OGs (HOGFasta output in OMA) of peptidases and peptidase inhibitors were found in the platyhelminths analysed (Supplementary Table S7 and File S4); however, only 85 OGs were present in at least 10 species (Supplementary File S5). The phylogenetic analysis was performed based on these 85 OGs. The multilocus phylogenetic tree of peptidases showed the same topology as the phylogenetic trees of the BUSCO and OMA OGPs (Figure 1 and Figure 4A). The monopisthocotyleans were clustered with high support (100%) and had a sister-group relationship with cestodes, although with low support (Bs = 48%). The polyopisthocotyleans formed a well-supported group (100%) and a well-supported sister-group relationship with trematodes (Bs = 97%). Cestodes and trematodes also formed their respective well-supported monophyletic groups (Bs = 100%). Details of the OGs annotation are shown in Supplementary Tables S8 and S9.

#### 3.1.3. Network Analysis and Hierarchical Grouping Analysis in Principal Components of Orthologous Groups

To identify the OGs most related to each class of platyhelminths, network analysis and a hierarchical grouping analysis in principal components (HCPC) was performed using the peptidases and peptidase inhibitors. The network analysis showed four clusters; a first cluster was formed by monopisthocotyleans, a second by cestodes, a third by both trematodes and polyopisthocotyleans, and a fourth by free-living species (Supplementary Figure S4). The network analysis clusters 134 OGs with monopisthocotyleans, 96 with cestodes, 176 trematodes and polyopisthocotyleans, and 125 OGs with free-living platyhelminths (Supplementary Table S10). 

The HCPC analysis showed a similar clustering as that observed in the network analysis (Figure 4B). The cluster of monopisthocotyleans was associated with 27 OGs, cestodes with 54 OGs, trematodes + polyopisthocotyleans with 72 OGs, and free-living species with 114 OGs. These associations were significant and positive (Supplementary Table S11). OGs associated with each group in both the network and HCPC analysis are shown in Supplementary Table S12.

**Figure 5:**
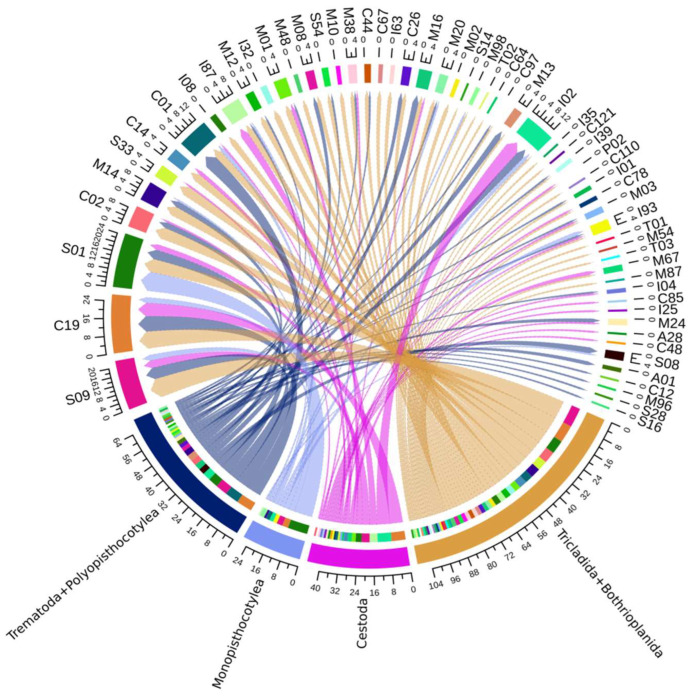
Circus chart representing the OGs of peptidase and inhibitor families that most contribute to the formation of groups in the HCPC and network analyses.

The main families that contributed to the formation of the four clusters in the network and HCPC analyses were C19, S09, S01, C02, C01, S01, I02, and I87 (Figure 5 and Supplementary Table S12). Particularly, the family I02 was important for the grouping of cyclophyllidean cestodes, the subfamily C01A for trematodes + polyopisthocotyleans, the subfamily S01C for monopisthocotyleans, and the subfamilies S09X and C02A for the free-living species used. Given their biological importance for neodermatan parasites [23,67,68], the C01A (cathepsin) and S01C (cercarial elastase) peptidases were further classified (see below).

### 3.2. Mutation Rate in Secreted and Non-Secreted Peptidases

#### 3.2.1. Single-Copy Orthologous Groups vs. Multiple-Copy Orthologous Groups

While Ka was higher in the multiple-copy OGs than in single-copy OGs (Supplementary Figure S5A), Ks was similar between OGs of single-copy and multiple-copy OGs (Supplementary Figure S5B). The lowest Ka and Ks in both single-copy and multiple-copy OGs were observed between cestode species (Supplementary Figure S6), although this may be because the three species of cestodes belong to the same order. The highest Ka in multiple-copy OGs was observed between monopisthocotylean species (Supplementary Figures S5A and S6), while the highest Ks in single-copy OGs were similar between platyhelminths, except in cestodes. All values of Ka and Ks are presented in Supplementary Table S13.

#### 3.2.2. Single-Copy Secreted Protein Orthologous Groups vs. Single-Copy Non-Secreted Proteins Orthologous Groups 

**Figure 6:**
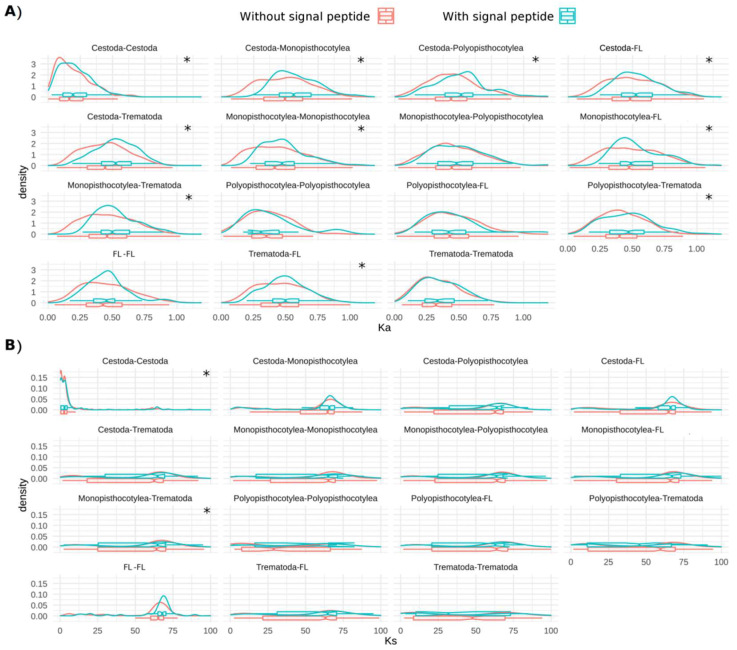
The distribution of (**A**) Ka and (**B**) Ks in single-copy OGs of peptidases and inhibitors of platyhelminths with/without predicted signal peptides. FL indicate the free-living platyhelminths *Bothrioplana semperi* and *Schmidtea mediterranea*. * indicates *p* < 0.05.

The analyses of secreted and non-secreted proteins were performed in both single-copy and multiple-copy OGs. Ka was higher in secreted proteins than in non-secreted proteins in most paired comparisons (Figure 6A). Ks in single-copy OGs was similar between secreted proteins and non-secreted proteins (Figure 6B) and between parasitic and free-living species. 

#### 3.2.3. Multiple-Copy Orthologous Groups Secreted Proteins vs. Multiple-Copy Orthologous Groups Non-Secreted Proteins

**Figure 7:**
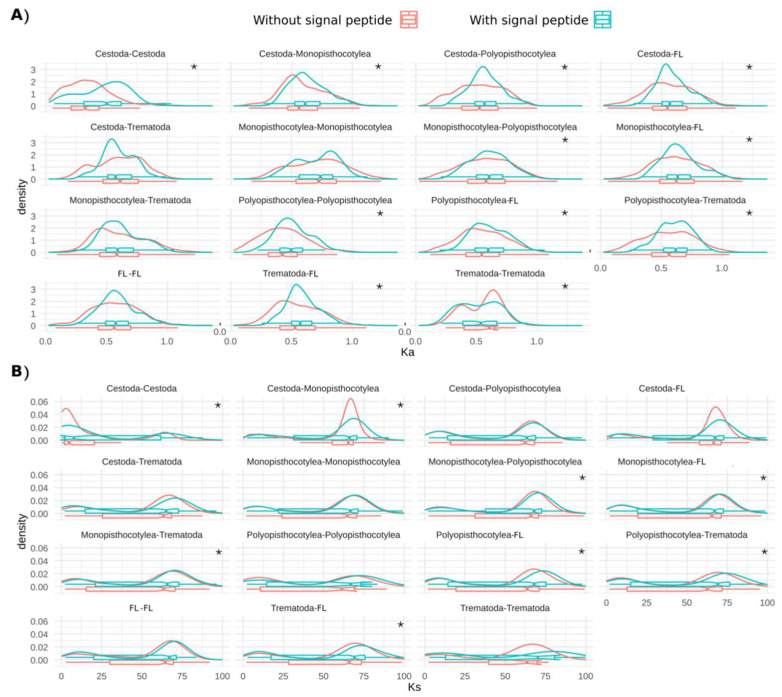
The distribution of (**A**) Ka and (**B**) Ks in multiple-copy OGs of peptidases and inhibitors of platyhelminths with/without predicted signal peptides. FL indicate the free-living platyhelminths *Bothrioplana semperi* and *Schmidtea mediterranea*. * indicate *p* < 0.05.

Ka and Ks in multiple-copy OGs were higher in secreted proteins than in non-secreted proteins in most paired comparisons (*p*-value < 0.05) (Figure 7). The comparison between trematode species was the only one that showed a Ka higher in non-secreted proteins than in secreted proteins (Figure 7A).

### 3.3. Classification of the C01A Peptidase Subfamily and S2 Active Subsite Residues

Members of the C01A papain-like peptidases were classified into two clades and three subclades as follows: subclade 1.1, formed by putative Cathepsin L proteins; subclade 2.1, formed by putative Cathepsin B proteins; and subclade 2.2, formed by putative dipeptidyl-peptidase proteins (Cathepsin C) (Table 2, Supplementary Table S14 and Figure S7). One subgroup within the cathepsin L (subclade 1.1) was formed only by peptidases of neodermatans. 

The specific arrangement of amino acids that create the S2 subsite within the active site of cathepsin peptidases largely determines the specificity of the enzymes [69,70]. Studies using functionally active recombinant molecules showed that changes at residue positions 67, 157, and 205 (papain numbering) had the most significant impact on substrate specificity in *F. hepatica* cathepsins L [66,71,72]. Thus, the amino acid composition of the S2 subsite, at these three positions, was compared among the various platyhelminth cathepsin L sequences (Supplementary Figure S8 and Table S15). Leucine was the most represented amino acid at positions 67 and 157 in all species examined, while Methionine occurred most frequently at position 205 in all species, except *E. nipponicum*, *F. hepatica*, and *N. melleni*. 

We identified nine cathepsin L belonging to cestodes, trematodes, and monopisthocotyleans, which contained a tyrosine at position 67 (TASs01205g12006m00001, EmuJ_000989200.1, TASs00007g01862m00001, HmN_000323300, Smp_187140.1, TASs00112g07689m00001, maker-scf7180006948404-augustus-gene-0.32-mRNA-1, TRINITY_DN2691_c0_g1_i1__g.61367, and TRINITY_DN5043_c0_g1_i3__g.56686). Of these, the proteins belonging to cestodes and trematodes showed the same amino acids at positions 67 and 157 (tyrosine and leucine, respectively) that occur in the FhCL2 cathepsin (Supplementary Table S15). Additionally, we identified three cathepsin L sequences belonging to monogeneans (E_nip_trans_37948_m.257852, TRINITY_DN10437_c1_g1_i2__g.43598, and TRINITY_DN3178_c0_g1_i10.p1) with the same amino acid distribution in the S2 subsite as FhCL5 (leucine at all three positions).

### 3.4. Classification of the S01C Peptidase Subfamily

The subfamily S01C was classified into two clades (clades 1 and 2) (Table 2, Supplementary Figure S9 and Table S14). The peptidases of clade 1 were formed by proteins of pathogenic neodermatans and clustered with the cercarial elastase peptidase (Smp_330280.1 = MER0003620) of *S. mansoni*. This group was overrepresented by monopisthocotylean proteins and lacked members from cestodes and free-living species. The clade 2 was comprised of proteins from all platyhelminth classes.

## 4. Discussion

In the present study, phylogenetic analyses based on OGPs from BUSCO and OMA, and peptidase OGs were performed to investigate the evolutionary relationships within Neodermata. Our dataset included 11 species of Neodermata, with members of both subclasses of Monogenea (Polyopisthocotylea and Monopisthocotylea), which were generally underrepresented in previous studies. The most important results at the phylogenetic level were the following: (1) It is corroborated again that Monogenea is not a monophyletic group because its subclasses are nested in different clades; (2) Monopisthocotylea and Cestoda were grouped in the same clade; and (3) Polyopisthocotylea and Trematoda were also grouped in the same clade. These findings are discussed below.

### 4.1. Non-Monophyly of Monogenea

The non-monophyly of the monogeneans has been the subject of discussion for many years, and this has intensified since molecular data began to be used to infer its phylogenetic position. In principle, the use of different sources of information (e.g., morphological vs. molecular), of different molecular markers (e.g., nuclear vs. mitochondrial and DNA vs. amino acid sequences), of different loci (e.g., a single gene vs. multigenes), of total evidence (i.e., morphology + molecular), and of different taxa included in the analyses (e.g., exclusion of some groups of monogeneans) has led to the formulation of inconsistent phylogenetic hypotheses (Supplementary Table S16).

The pioneering studies of Lambert [73], Brooks et al. [74], Ehlers [75], Rohde [76], Justine [8], and Boeger and Kritsky [7], offered a first approximation of the position and phylogenetic relationships of Monogenea, using morphological characters. However, it was Justine [77] who questioned the monophyly of Monogenea, based on spermatological analysis which suggested there was no synapomorphy that supported the monophyly of the group. Whilst surprising, this finding was also corroborated by the first phylogenetic analyses to use DNA sequences [78,79,80,81], although a subsequent molecular study obtained the opposite result, that Monogenea is monophyletic [42]. A later study combining molecular and morphological characters again suggested the monophyly of Monogenea [2], and another phylogenetic study with only morphological characters suggested that Monopisthocotylea and Polyopisthocotylea share the following synapomorphies: (1) larva with three zones ciliated; (2) two pairs of pigmented eyes in larvae and adults, with retention of the number and distribution of larval eyes in the adult; (3) a pair of ventral anchors; and (4) an egg filament [5,6]. Despite this, the monophyly of Monogenea continues to be questioned based on the increasing incorporation of new molecular data, such as more and new loci (ribosomal and mitochondrial genes), complete mitochondrial genomes (mitogenomes), genomes, and transcriptomes [3,4,10,82]. An alternative hypothesis that tries to explain why Monogenea cannot be consistently resolved as a monophyletic group is that once the Polyopisthocotylea separated from Monopisthocotylea, they had a rapid molecular divergence, accumulating a large number of mutations, which ended in homoplasies that can generate noise in phylogenetic inference [83]. However, studies using large DNA and amino acid sequence datasets from different genomic regions of the parasites, such as the one presented in this study, are convincing in obtaining independent and well-supported clades of the monogeneans analysed. Although the most recent studies (including ours) do not have a wide representation of species of both subclasses of monogeneans, it is clear that even with few taxa, large sets of molecular data indicate that Monopisthocotylea and Polyopisthocotylea do not nest in the same clade, which is consistent with our findings using OGPs. Therefore, all the studies that agree with the non-monophyly of Monogenea support the idea that the Monopisthocotylea and the Polyopisthocotylea do not share a recent common ancestor (despite similarities in their life cycle and in some morphological characteristics), and that they diverged from different ancestors that were more closely related to the other groups of Neodermata.

### 4.2. Monopisthocotylea + Cestoda

In the present study, the monophyly of Monopisthocotylea plus Cestoda was only well supported with OMA OGPs; nonetheless, the high support was not observed when the analysis was repeated using less data. In addition, our tree topology test based on BUSCO OGPs showed that a scenario in which Cestoda and Trematoda are grouped in a clade cannot be rejected. Other studies based on molecular data have already suggested a sister relationship between monogeneans and cestodes [10,12], which was also indicated by the presence of cercomers, or hooks, on the posterior end of larvae [84]. However, Lockyer et al. argued that the grouping of Monogenea with Cestoda by the cercomers is unreliable [6], and phylogenetic analyses using rRNA genes or mitochondrial genomes did not support this sister-group relationship [3,6].

Additionally, another morphological characteristic shared between monopisthocotyleans and cestodes (at least with *Gyrocotylidea*) is the presence of anterior nephridiopores [12,85]. It is interesting that our data provide some evidence in favour of a sister-group relationship between Cestoda and Monopisthocotylea when using peptidases. Because rRNA genes or mitochondrial genomes (used in conflicting studies) are sensitive to sequence alignment methods and are subject to rapid substitution rates [12], it is possible that peptidases may have value as phylogenetic markers in future studies.

### 4.3. Polyopisthocotylea + Trematoda

The monophyly of Polyopisthocotylea and Trematoda found in this study was also detected in the phylogenetic analysis of 202 single-copy OGPs of a number of helminth species (although this study did not include monopisthocotylean species) [82] and in a previous study using 28S DNA sequences [81]. It should also be noted that in the latter study, the clade Monopisthocotylea + Cestoda was also obtained. Our tree topology test based on BUSCO OGPs indicated that different scenarios are possible; nonetheless, the monophyly of Polyopisthocotylea and Trematoda was well supported by peptidase OGs and OMA OGPs, even when a relatively smaller dataset was used.

Trematoda have a variety of diets, including host blood and epithelia, with digestion in the gut being largely extracellular, while the Polyopisthocotylea feed on blood and have intracellular digestion [83]. Previously it had been suggested that the ancestor of the Trematodes could have been a Polyopisthocotylea-like sanguinivore, because a polyopisthocotylean was the sister species of the clade formed by Trematoda + Cestoda [3]. However, our findings do not support this hypothesis, because Polyopisthocotylea was grouped as the sister-group of Trematoda (and Cestoda with Monopisthocotylea), where it is also possible that the hypothetical ancestor of this clade could have had a diet different from blood. Whilst the adaptive changes involved in feeding were likely important in driving the evolution of Neodermata, better ancestral reconstructions based on diet are required (e.g., inclusion of genomic data from other important groups, such as Aspidosgastrea, although this is currently lacking).

In terms of morphology, there are currently no conclusive morphological synapomorphies that support this clade. However, Littlewood et al. [2] proposed that Trematoda shares with Polyopisthocotylea a neodermatan type flame bulb accompanied by a protonephridial capillary with septate junction (see characters 14 and 19 in the matrix of morphological characters in the appendices of Littlewood et al. [2]). However, although this characteristic is absent in Cestoda, within Neodermata it can also be found in some Monopisthocotylea, so it is not entirely clear if it is a plesiomorphy. Therefore, it is necessary to continue exploring helminth morphology to find possible synapomorphies in this clade that support it.

In the present study, the lack of evidence of a sister-group relationship between Trematoda and Cestoda agrees with the absence of any known morphological apomorphies for the Cestoda–Trematode clade [6,12], a clade supported by other studies [1,3]. Likewise, the molecular evidence does not always support a sister-group relationship between Cestoda and Trematoda [82]. As mentioned above, it is likely that cestodes and trematodes diverged independently, therefore the absence of apomorphies is to be expected. This independent divergence could explain the difference in life history traits, such as the use of intermediate hosts (molluscs in Trematoda and mainly crustaceans in Cestoda) or feeding strategies [11].

### 4.4. Peptidases in Neodermata

The similarity of topologies of the phylogenetic trees based on peptidases and BUSCO and OMA OGPs suggest that these proteins may shed some light on the evolutionary relationships of Neodermata. The peptidases and peptidase inhibitors play an important role in the feeding processes of the neodermatans, with parasite-derived peptidases being particularly important in host tissue digestion [24,86]. Indeed, the diversification of these proteins is suggested to have contributed to the success of the parasitic lifestyle [87]. According to our network and HCPC analyses, the family C01 (papain family) was the most important for the grouping of trematodes + polyopisthocotyleans, I02 (Kunitz-BPTI family) for cestodes, and S01 (chymotrypsin family) for monopisthocotyleans. Because only hematophagous species of trematodes and polyopisthocotyleans were included in this analysis, the question remains of whether the C01 family is also important for non-hematophagous species. The expansions of C01 and S01 could be linked with adaptation to a parasitic lifestyle. For example, the serine peptidases, members of the subfamily S01, may be used by the monopisthocotyleans to digest host tissue during feeding [88]. Peptidases of the C01 family are used by trematodes to penetrate the host and migrate to specific organs, with the cathepsins B, F, and L among the most studied [21,27,68]. Similarly, the proteinase inhibitors perform important functions for the survival of the parasite in the host because they are responsible for inhibiting host enzymes or manipulating host immune responses [24,89]. Members of the also expanded I02 inhibitor family have anticoagulant and anti-inflammatory function [24] and include the Kunitz-type inhibitors that in cestodes suppress the proteolytic activity of their host [89,90].

The present study suggests that peptidases/inhibitors with multiple copies (and those secreted by helminths into host tissues) are under positive selection pressure, which could contribute to the expansion of certain families such as C01, C19, I02, and S01 (see Figure 2 and Supplementary Figures S7 and S9). As these participate in the host–parasite interaction [22,23,27], it is likely that natural selection is acting on them. Indeed, Li et al. [91] observed that the secreted proteins of bacteria and fungi evolve faster than non-secretory proteins, which they suggested is due to selective pressure helping to shape proteins with particular biochemical adaptations to the environment.

Members of the C01 (papain) cysteine peptidases, one of the most abundant peptidase families found in the present study, perform functions related to nutrition, tissue invasion, and immune system evasion in helminths [25]. Recent studies have shown that these peptidases, particularly cathepsins B, C, and L, are secreted by the monogenean *E. nippoonicum* [30], with cathepsin L participating in the digestion of host blood proteins [92]. Likewise, the trematode *F. hepatica* uses distinct cathepsin L enzymes to degrade host haemoglobin (FhCL1) and to penetrate the host duodenum/migrate through the liver parenchyma due the unique collagenolytic activity of certain family members (FhCL2 and FhCL3) [27,71,93]. Like other cysteine peptidases, the different substrate preferences of the *Fasciola* cathepsin L family members are conferred by the specific arrangement of amino acids that create the S2 subsite [66,69,70]. For instance, FhCL1 (S2 subsite: Leu^67^, Val^157^ and Leu^205^) prefers to cleave leucine and phenylalanine-containing substrates (haemoglobin is particularly rich in these) so it may have specifically evolved to facilitate blood feeding [93]. FhCL2 (S2 subsite: Tyr^67^, Leu^157^ and Leu^205^) and FhCL3 (S2 subsite: Trp^67^, Val^157^ and Val^205^) both accept proline-containing substrates and can digest collagen, thus aiding in host tissue degradation [66,71,72]. It is noteworthy that the majority of cathepsins L from the varied taxonomic groups studied here displayed an S2 subsite topology similar to FhCL1 (i.e., Leu^67^, Val^157^) which could suggest roles for these enzymes in blood feeding. However, FhCL1 has activity beyond haemoglobin degradation (reviewed by Cwiklinski et al. [68]), so this S2 subsite arrangement would conceivably allow cleavage of a variety of host macromolecules depending on host niche and mechanism of nutrient acquisition. Nine cathepsin L sequences displayed an S2 subsite arrangement similar to FhCL2 (i.e., Tyr^67^). Collagen is a major component of vertebrate connective tissue so the possible collagenase activity of these enzymes would aid parasite migration through host tissues and, in the case of monopisthocotyleans, help degrade the epidermis. In the present study, the phylogenetic relatedness of *F. hepatica* cathepsins L with those of other taxa of Neodermata suggest that these peptidases are important for the adaptation and evolution of neodermatans more generally.

The expansion of the S01C subfamily, mainly in most of the monopisthocotyleans studied here, indicates that these peptidases are particularly important for this group of monogeneans. Members of S01C have essential roles in protein digestion and pathogen invasion [22]. Ingram et al. [67] reported that cercarial elastase in *S. mansoni* (a S01C family member) is essential for host skin penetration and that the expansion of this group of peptidases may imply the acquisition of new functions related to host invasion. Future experimental studies are required to investigate the role of the S01C peptidases identified in this study, during infection by species such as *R. viridisi*, *G. salaris*, *N. melleni*, and *E. nipponicum* [94,95,96].

## 5. Concluding Remarks

In summary, based on putative OGPs, this study provides evidence in favour of the monophyly of polyopisthocotyleans + trematodes, which has not been discussed in previous studies. In addition, we detected a possible sister-group relationship between monopisthocotyleans and cestodes, although its statistical support was low. To the best of our knowledge, it is the first study to include peptidases in order to clarify the evolutionary relationships within Neodermata. We observed that multicopy and parasite-secreted peptidases/inhibitors were subject to higher selective pressure than single-copy and non-secreted peptidases/inhibitors, which could explain the expansion of some families such as C01, C19, I02, and S01, involved in host–parasite interaction. Whilst not definitive, we hope that our study will stimulate further research and debate on the evolution of Neodermata.

## Figures and Tables

**Table 1:** Tree topological test for four evolutionary scenarios of neodermatans, based on OGPs of two datasets (BUSCO and OMA).

Scenario	Constrained Tree	logL	deltaL	bp-RELL	*p*-KH	*p*-SH	c-ELW	*p*-AU	Reference
BUSCO OGPs									
1	((Monopisthocotylea, Cestoda), (Polyopisthocotylea, Trematoda))	−504,202.9113	0	0.941+	0.943+	1+	0.938+	0.947+	Present study
2	((Monopisthocotylea, Polyopisthocotylea), (Cestoda, Trematoda))	−504,265.679	62.768	0.0137−	0.0567+	0.0567+	0.0205−	0.0542+	[6]
3	(((Monopisthocotylea, Polyopisthocotylea), Cestoda), Trematoda)	−504,265.679	62.768	0.0208−	0.0567+	0.0567+	0.0205−	0.0538+	[42]
4	(((Trematoda, Cestoda), Polyopisthocotylea), Monopisthocotylea)	−504,265.679	62.768	0.0248+	0.0567+	0.0567+	0.0205+	0.0534+	[3]
OMA OGPs									
1	((Monopisthocotylea, Cestoda), (Polyopisthocotylea, Trematoda))	−2,015,132.409	0	0.987+	0.987+	1+	0.986+	0.986+	Present study
2	((Monopisthocotylea, Polyopisthocotylea), (Cestoda, Trematoda))	−2,015,328.208	195.8	0.0029−	0.0131−	0.0131−	0.00454−	0.0142−	[6]
3	(((Monopisthocotylea, Polyopisthocotylea), Cestoda), Trematoda)	−2,015,328.208	195.8	0.0044−	0.0131−	0.0131−	0.00454−	0.0142−	[42]
4	(((Trematoda, Cestoda), Polyopisthocotylea), Monopisthocotylea)	−2,015,328.208	195.8	0.0062−	0.0131−	0.0131−	0.00454−	0.0144−	[3]

deltaL: logL difference from the maximal logl in the set. bp-RELL: bootstrap proportion using RELL method [43]. *p*-KH: *p*-value of one-sided Kishino–Hasegawa test [44]. *p*-SH: *p*-value of Shimodaira–Hasegawa test [45]. c-ELW: Expected Likelihood Weight [46]. *p*-AU: *p*-value of approximately unbiased (AU) test [47]. Plus signs denote the 95% confidence sets. Minus signs denote significant exclusion. All tests performed 10,000 resamplings using the RELL method.

**Table 2:** Main members of the C01A and S01C subfamilies are classified in the phylogenetic analysis.

	C01A Subfamily			S01C Subfamily
Species	Cathepsin B	Cathepsin C	Cathepsin L	Cercarial Elastase
*E. multilocularis*	2	0	4	0
*H. microstoma*	2	0	4	0
*T. asiatica*	3	0	7	0
*G. salaris*	3	3	7	19
*N. melleni*	10	1	5	37
*R. viridisi*	2	1	3	8
*S. longicornis*	6	2	14	0
*E. nipponicum*	2	5	12	1
*P. xenopodis*	6	3	7	0
*F. hepatica*	17	0	16	1
*S. mansoni*	6	1	6	5
*B. semperi*	9	1	16	0
*S. mediterranea*	2	2	20	0
Total	70	19	121	71

## Data Availability

All data files generated in this study are available in the Supplementary Materials.

## Supplementary Materials

The following supporting information can be downloaded at: https://www.mdpi.com/article/10.3390/tropicalmed8010059/s1, Figure S1: Constrained trees constructed of 13 platyhelminth species, based on single-copy BUSCO OGPs and simply OMA OGPs; Figure S2: Number of peptidases (A) and peptidase inhibitors (B) identified in the selected platyhelminth species; Figure S3: Number and percentage of peptidase classes in each flatworm species; Figure S4: Formation of clusters of peptidases/inhibitors OGs and platyhelminth species in network analysis; Figure S5: Distribution of Ka and Ks in peptidases and inhibitors of platyhelminths. (A) Ka was higher in multiple-copy OGs than in single-copy OGs. (B) Ks was similar between single-copy OGs and multiple-copy OGs; Figure S6: Distribution of Ka and Ks in peptidases and inhibitors of platyhelminths, with the center of each legend positioned at the highest peak of the distribution; Figure S7: Phylogenetic analysis of the C01A peptidase subfamily. The alignment of the sequences was used to identify the S2 subsite residues belonging to cathepsins L (position 67, 157, and 205) in platyhelminths; Figure S8: Frequency of the amino acids comprising the S2 subsite (positions 67, 167 and 205) of cathepsins L belonging to the C01A subfamily in platyhelminths; Figure S9: Phylogenetic analysis of the S01C peptidase subfamily; Figure S10: Gene Ontology annotation of proteins used in the phylogenomic analysis; Figure S11: Number of shared peptidase families; Table S1: BUSCO OGPs of 13 platyhelminth species; Table S2: OMA OGPs of 13 platyhelminth species; Table S3: Peptidase and inhibitors identified in 13 platyhelminth species; Table S4: Number and percentage of peptidase classes in each flatworm species; Table S5: Number and percentage of peptidase families in each flatworm species; Table S6: Number of peptidases with predicted signal peptides in the 13 platyhelminth species analysed; Table S7: OMA OGs of peptidases of 13 platyhelminth species; Table S8: OGs of peptidases of 13 platyhelminth species used in phylogenetic analysis; Table S9: Number of OGs of peptidases by family used in phylogenetic analysis; Table S10: OGs of peptidases and inhibitors that most contribute to the formation of groups network analyses; Table S11: OGs of peptidases and inhibitors that most contribute to the formation of groups in the HCPC analyses; Table S12: Annotation of the OGs of peptidases and inhibitors that most contribute to the formation of groups in the HCPC analyses; Table S13: Ka and Ks of peptidases and inhibitors of platyhelminths; Table S14: Members of the C01A and S01C subfamilies that were classified in the phylogenetic analysis; Table S15: The S2 subsite residues belonging to cathepsins L (position 67, 157, and 205) identified in platyhelminths; Table S16: Phylogenetic results of Neodermata in other studies; File S1: Fasta files of the 137 BUSCO OGPs used in the phylogenetic analyses; File S2: Fasta files of the 479 OMA OGPs used in the phylogenetic analyses; File S3: Fasta files of the 3960 peptidases identified in the 13 species of platyhelminths; File S4: Fasta files of the 531 OGs of peptidases and peptidase inhibitors in the 13 species of platyhelminths; File S5: Fasta files of the 85 OGs of peptidases used in the phylogenetic analyses; Supplementary Results: Results about the identification of peptidases and inhibitors in 13 platyhelminth species.
