# Supplementary material for: Exploring Evolutionary Relationships within Neodermata Using Putative Orthologous Groups of Proteins, with Emphasis on Peptidases
Source: Trop Med Infect Dis. 2023 Jan 12;8(1):59. doi: 10.3390/tropicalmed8010059 (PMC9860727; doi:10.3390/tropicalmed8010059)
Supplement: Supplementary file 1 [file tropicalmed-08-00059-s001.zip › Supplement_R4/Suplementary FigureS1-S11.pdf]

Exploring Evolutionary Relationship within Neodermata using Putative Orthologous Groups of Proteins, with Emphasis on Peptidases

Víctor Caña-Bozada[1], Mark W. Robinson[2], David I. Hernández-Mena[3], Francisco N. Morales-Serna[4]

[1]Centro de Investigación en Alimentación y Desarrollo, A.C., Mazatlán 82112, Sinaloa, Mexico; [2]School of Biological Sciences, Queen's University Belfast, 19 Chlorine Gardens, Belfast, Northern Ireland, UK; [3]Centro de Investigación y de Estudios Avanzados, Instituto Politécnico Nacional, Unidad Mérida, Mérida 97310, Yucatán, Mexico; and [4]Instituto de Ciencias del Mar y Limnología, Universidad Nacional Autónoma de México, Mazatlán 82040, Sinaloa, Mexico

Corresponding author: Francisco N. Morales-Serna,

neptali@ola.icmyl.unam.mx

Supplementary Figures S1-S11

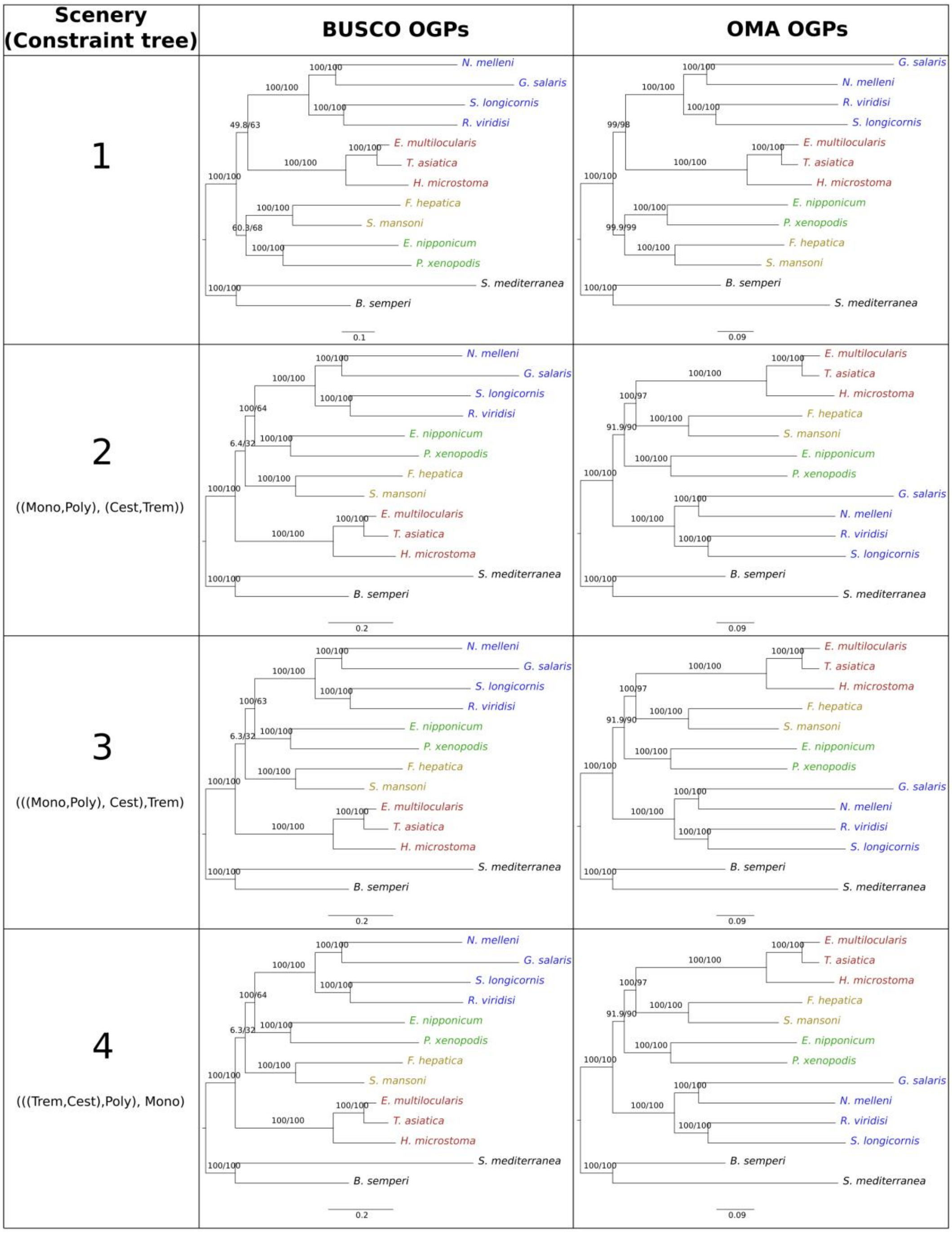

Figure S1: Constrained trees constructed of 13 platyhelminth species, based on single-copy BUSCO OGPs and simply OMA OGPs. Alternative scenarios obtained from the literature were used as guide (2, 3, 4) and the LG+F+R4 model. Values above represent SH-aLRT support (%). The free-living platyhelminths *Bothrioplana semperi*, belonging to the order Bothrioplanida, and *Schmidtea mediterranea*, belonging to the order Tricladida, were used as outgroups.

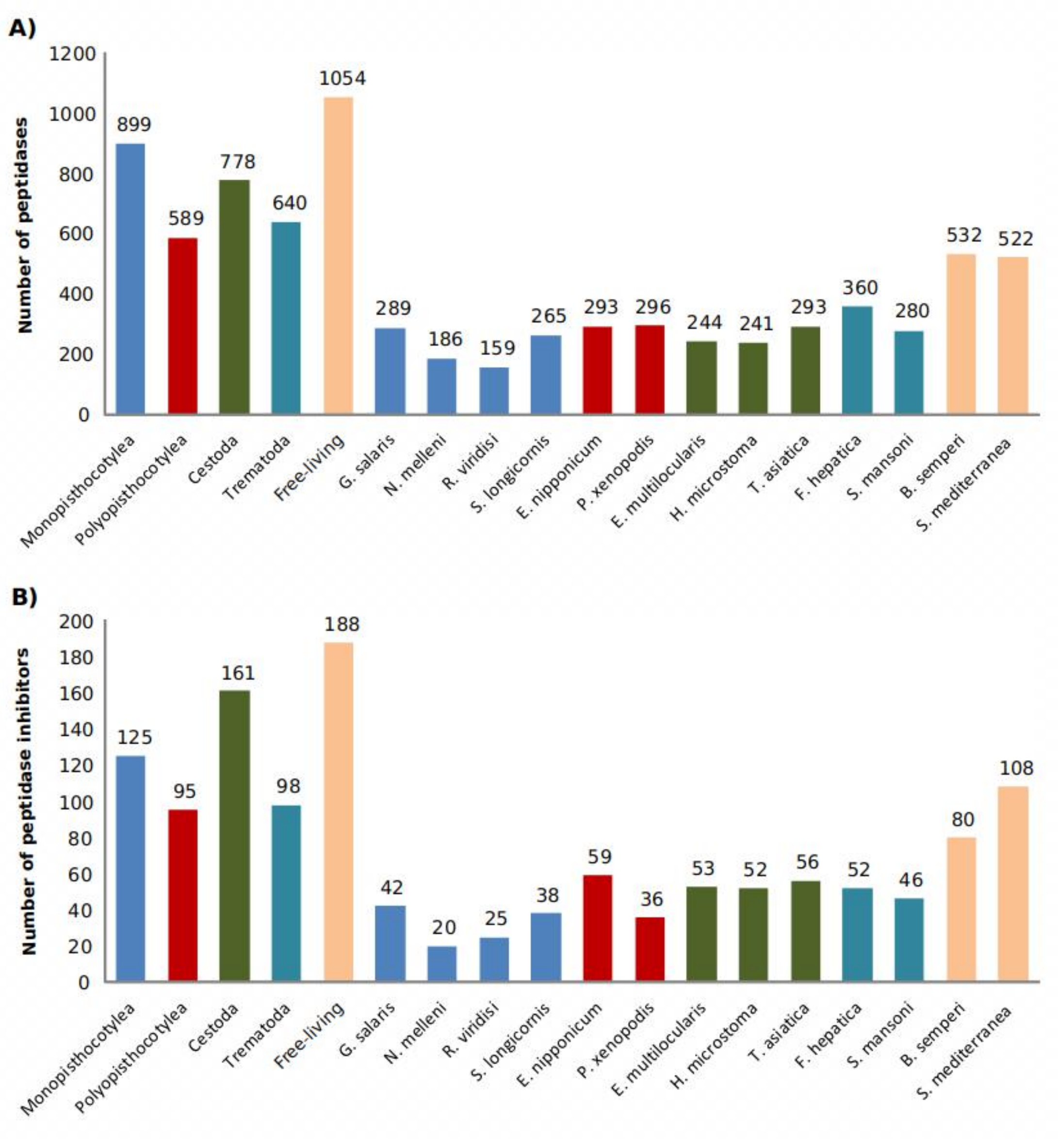

Supplementary Figure S2. Number of peptidases (A) and peptidase inhibitors (B) identified in the selected platyhelminth species.

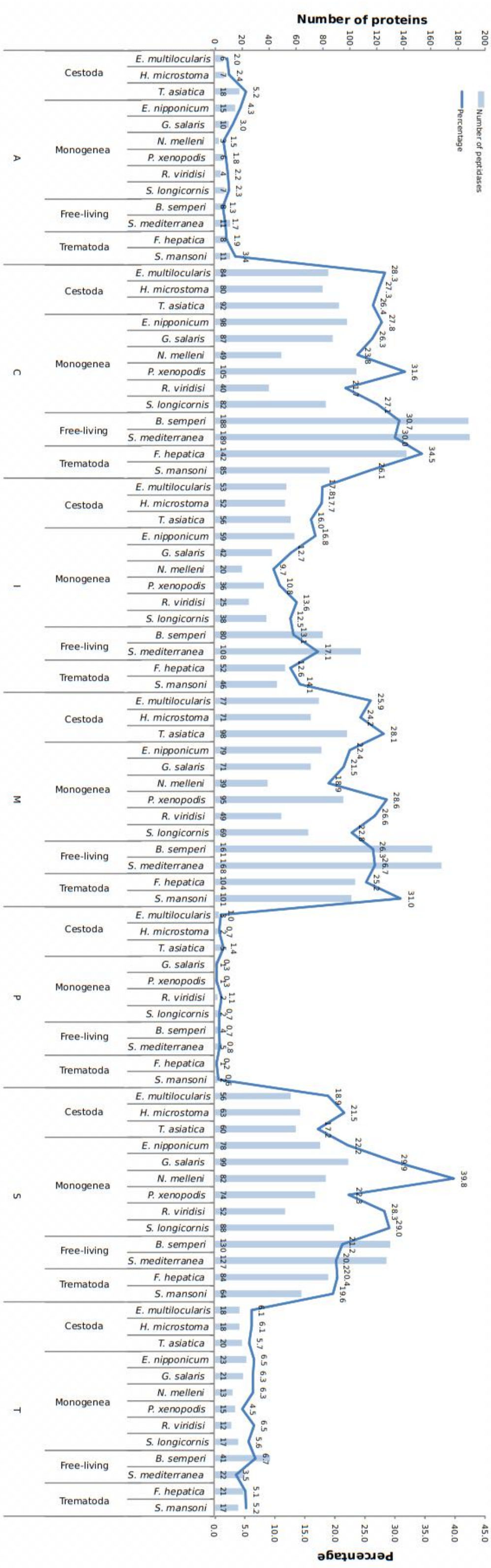

Supplementary Figure S3. Number and percentage of peptidase classes in each flatworm species.

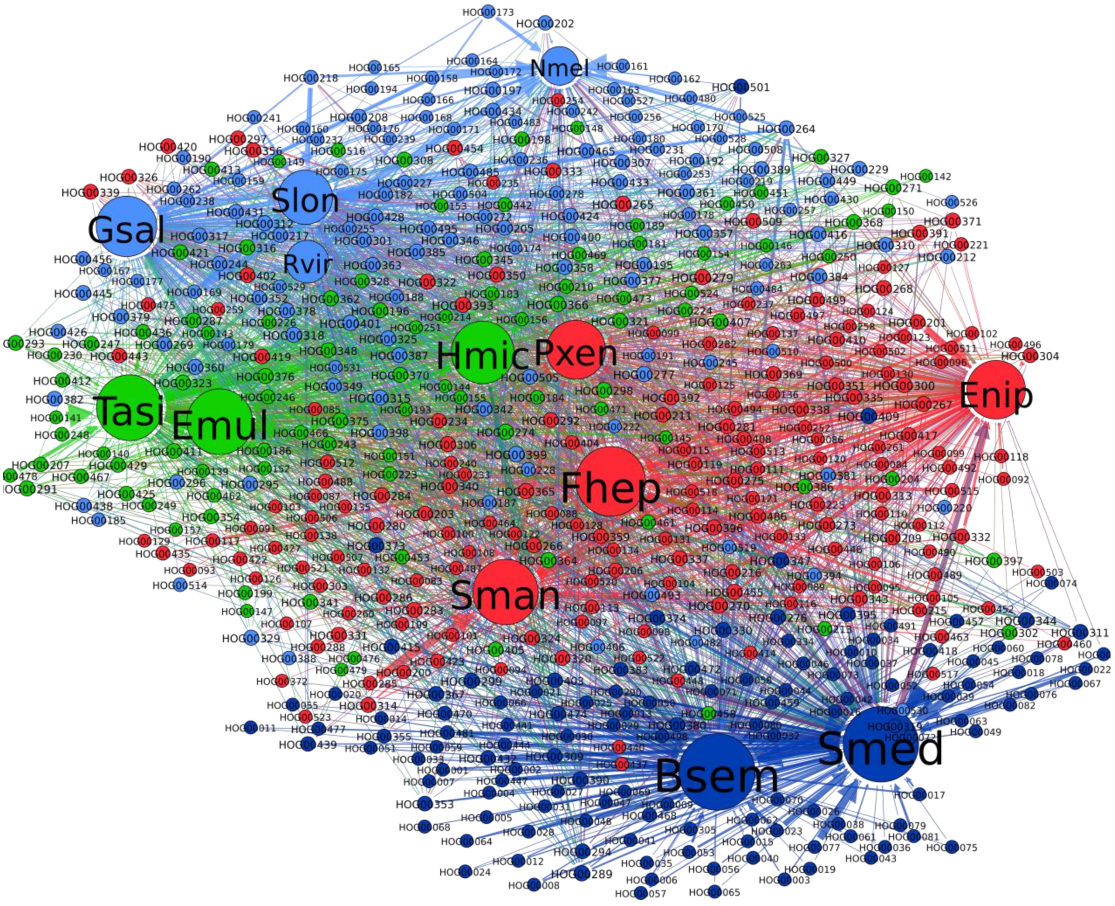

Supplementary Figure S4. Formation of clusters of peptidases/inhibitors OGs and platyhelminth species in network analysis. Monogenea Monopisthocotylea: Nmel, *Neobenedenia melleni;* Gsal, *Gyrodactylus salaris;* Slon, *Scutogyrus longicornis;* Rvir, *Rhabdosynochus viridisi.* Cestoda: Tasi, *Taenia asiatica;* Emul, *Echinococcus multilocularis;* Hmic, *Hymenolepis microstoma.* Monogenea Polyopisthocotylea: Enip, *Eudiplozoon nipponicum;* Pxen, *Protopolystoma xenopodis.* Trematoda: Fhe, *Fasciola hepatica;* Sman, *Schistosoma mansoni.* Free-living platyhelminths: Bsem, *Bothrioplana semperi;* Smed, *Schmidtea mediterranea.*

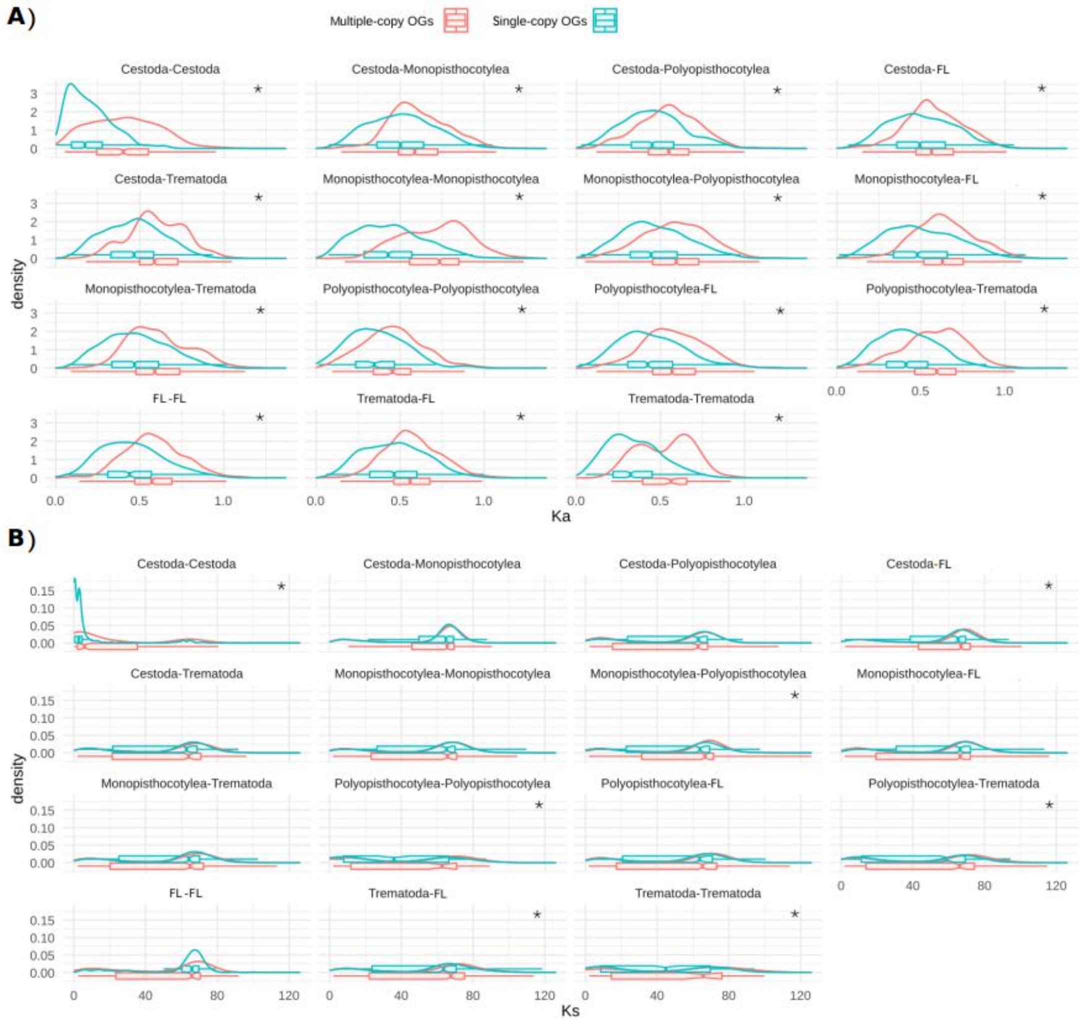

Supplementary Figure S5. Distribution of Ka and Ks in peptidases and inhibitors of platyhelminths. A) Ka was higher in multiple-copy OGs than in single-copy OGs. B) Ks was similar between single-copy OGs and multiple-copy OGs. FL, free-living platyhelminths. * indicate $p < 0.05$.

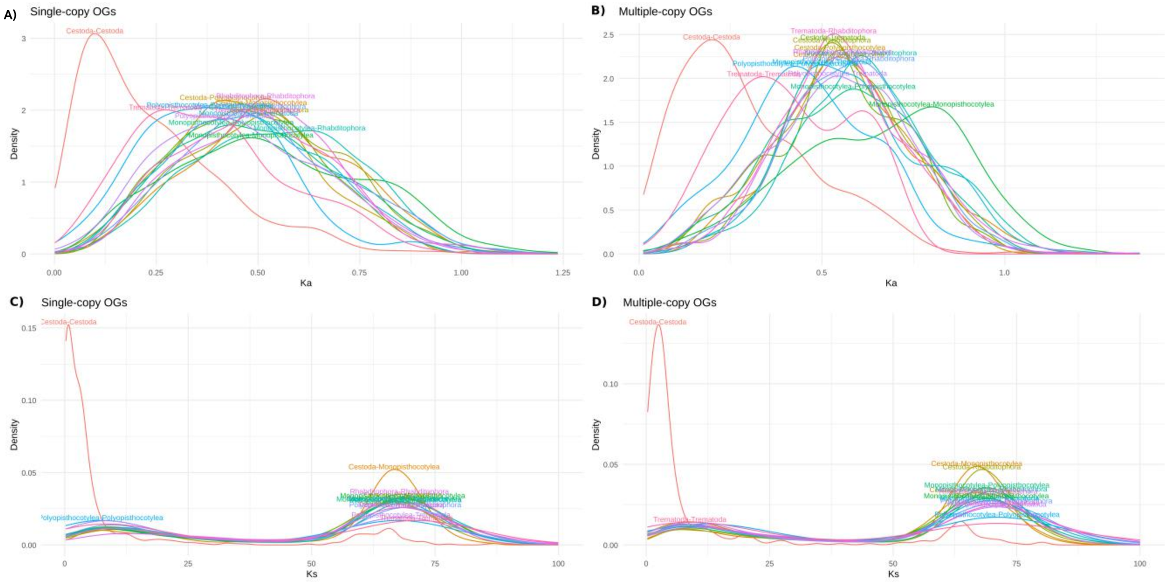

Supplementary Figure S6. Distribution of Ka and Ks in peptidases and inhibitors of platyhelminths, with the center of each legend positioned at the highest peak of the distribution. A) Distribution of Ka in single-copy OGs. B) Distribution of Ka in multiple-copy OGs. C) Distribution of Ks in single-copy OGs. D) Distribution of Ks in multiple-copy OGs.

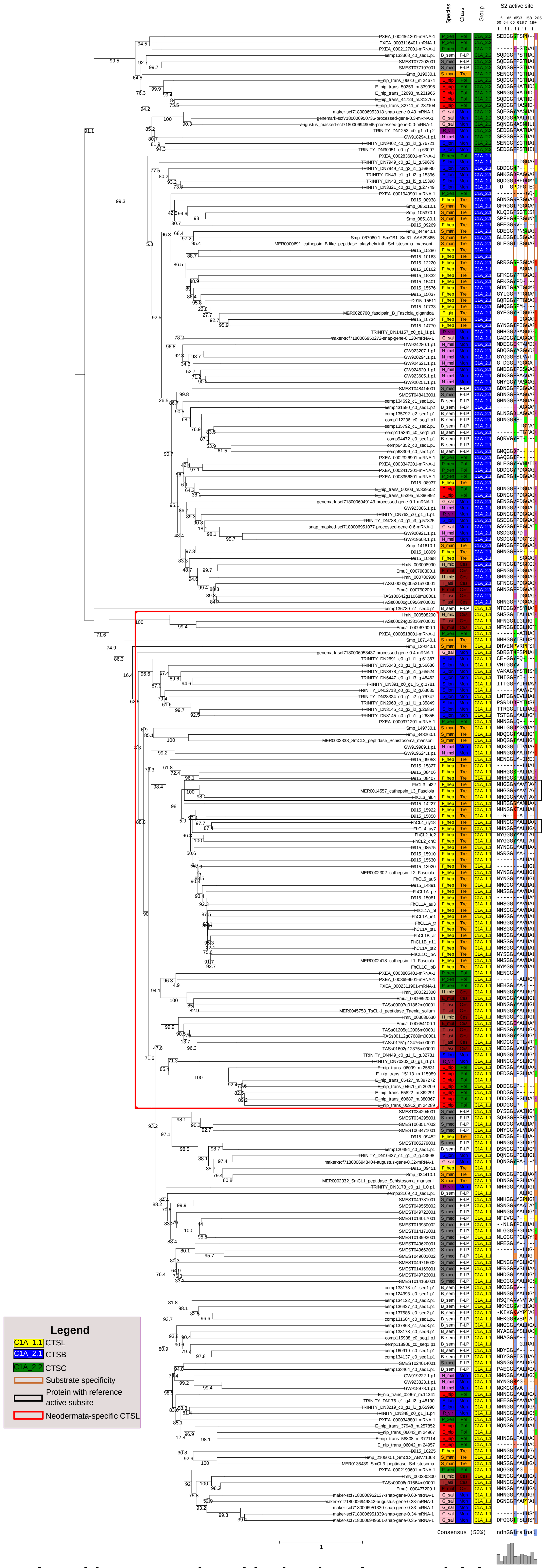

Supplementary Figure S7. Phylogenetic analysis of the C01A peptidase subfamily. The midpoint-rooted phylogenetic tree was constructed using 1000 replicates of the approximate likelihood ratio test (similar to the Shimodaira–Hasegawa test). The WAG+R6 model was implemented. The proteins of the MEROPS database were used as reference sequences. The alignment of the sequences was used to identify the S2 subsite residues belonging to cathepsins L (position 67, 157, and 205) (Stack et al., 2008; Robinson et al., 2008) in platyhelminths. Abbreviations: Mon, Monopisthocotylean; Pol, Polyopisthocotylea; Tre, Trematoda; Ces, Cestoda; F-LP, Free-living platyhelminths. N_mel, *Neobenedenia melleni*; G_sal, *Gyrodactylus salaris*; S_lon, *Scutogyrus longicornis*; R_vir, *Rhabdosynochus viridisi*; *t_asi, taenia asiatica*; E_mul, *Echinococcus multilocularis*; H_mic, *Hymenolepis microstoma*; E_nip, *Eudiplozoon nipponicum*; P_xen, *Protopolystoma xenopodis*; F_hep, *Fasciola hepatica*; S_man, *Schistosoma mansoni*; B_sem, *Bothrioplana semperi*; S_med, *Schmidtea mediterranea*. CTSL, cathepsins L; CTSB, cathepsins B; CTSC, cathepsins C.

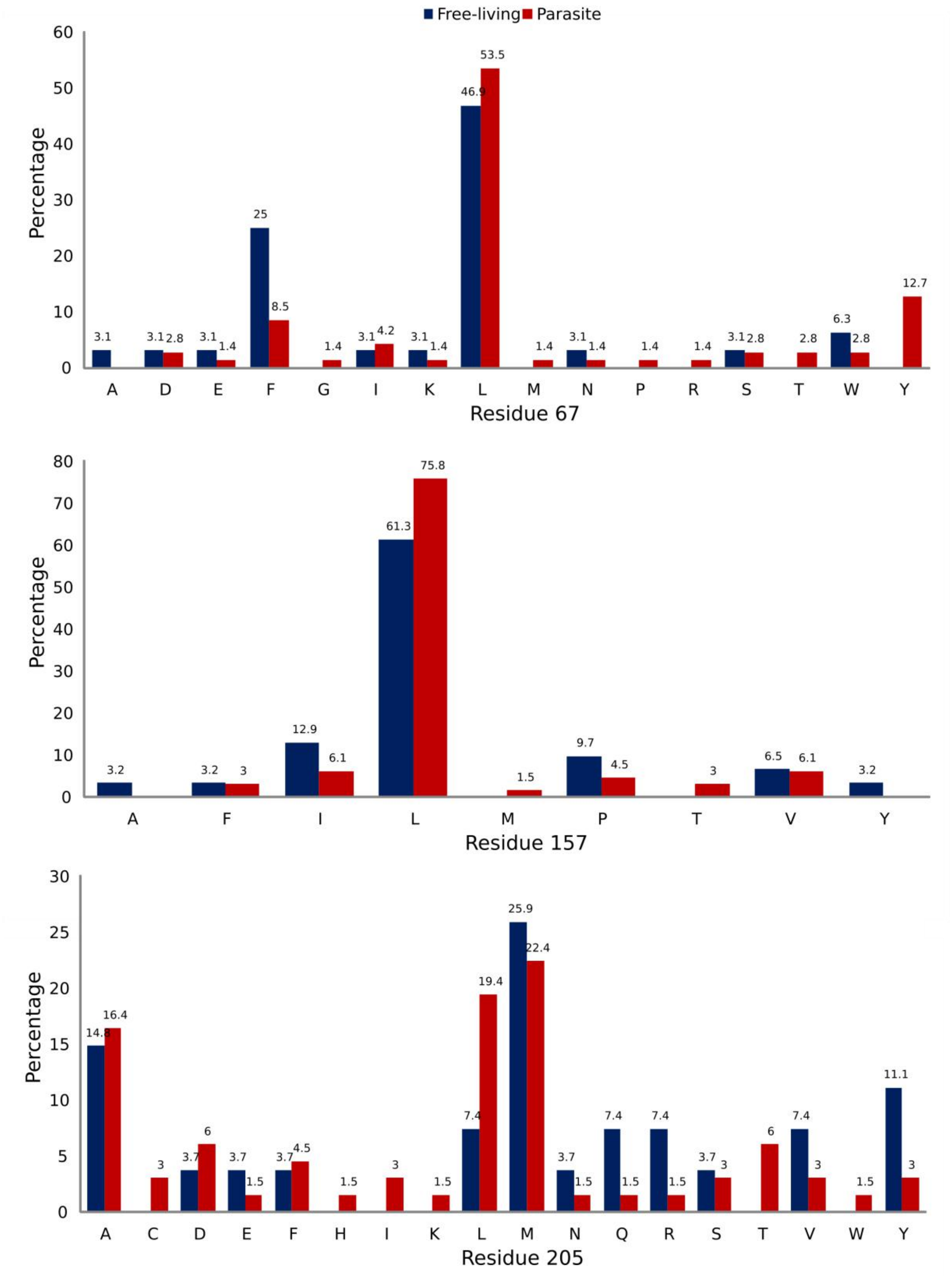

Supplementary Figure S8. Frequency of the amino acids comprising the S2 subsite (positions 67, 167 and 205) of cathepsins L belonging to the C01A subfamily in platyhelminths.

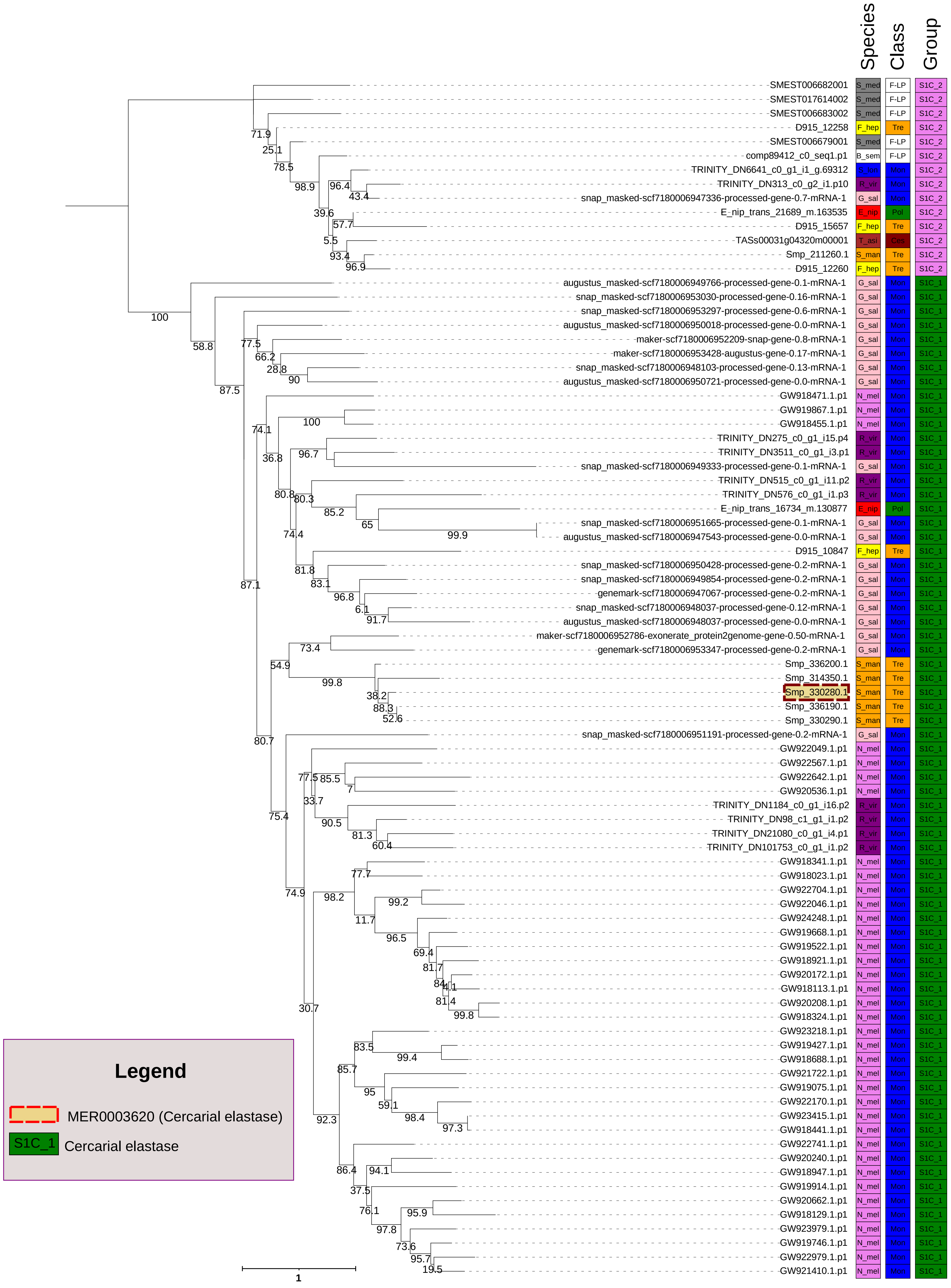

Supplementary Figure S9. Phylogenetic analysis of the S01C peptidase subfamily. The midpoint-rooted phylogenetic tree was constructed using 1000 replicates of the approximate likelihood ratio test (similar to the Shimodaira–Hasegawa test). The WAG+R5 model was implemented. The protein MER0003620 (Cercarial elastase) of the MEROPS database was used as reference sequences. Abbreviations: Mon, Monopisthocotylean; Pol, Polyopisthocotylea; Tre, Trematoda; Ces, Cestoda; CF-LP, Free-living platyhelminths. N_mel, *Neobenedenia melleni*; G_sal, *Gyrodactylus salaris*; S_lon, *Scutogyrus longicornis*; R_vir, *Rhabdosynochus viridisi*; *t_asi*, *taenia asiatica*; E_mul, *Echinococcus multilocularis*; H_mic, *Hymenolepis microstoma*; E_nip, *Eudiplozoon nipponicum*; P_xen, *Protopolystoma xenopodis*; F_hep, *Fasciola hepatica*; S_man, *Schistosoma mansoni*; B_sem, *Bothrioplana semperi*; S_med, *Schmidtea mediterranea*. CTSL, cathepsins L; CTSB, cathepsins B; CTSC, cathepsins C.

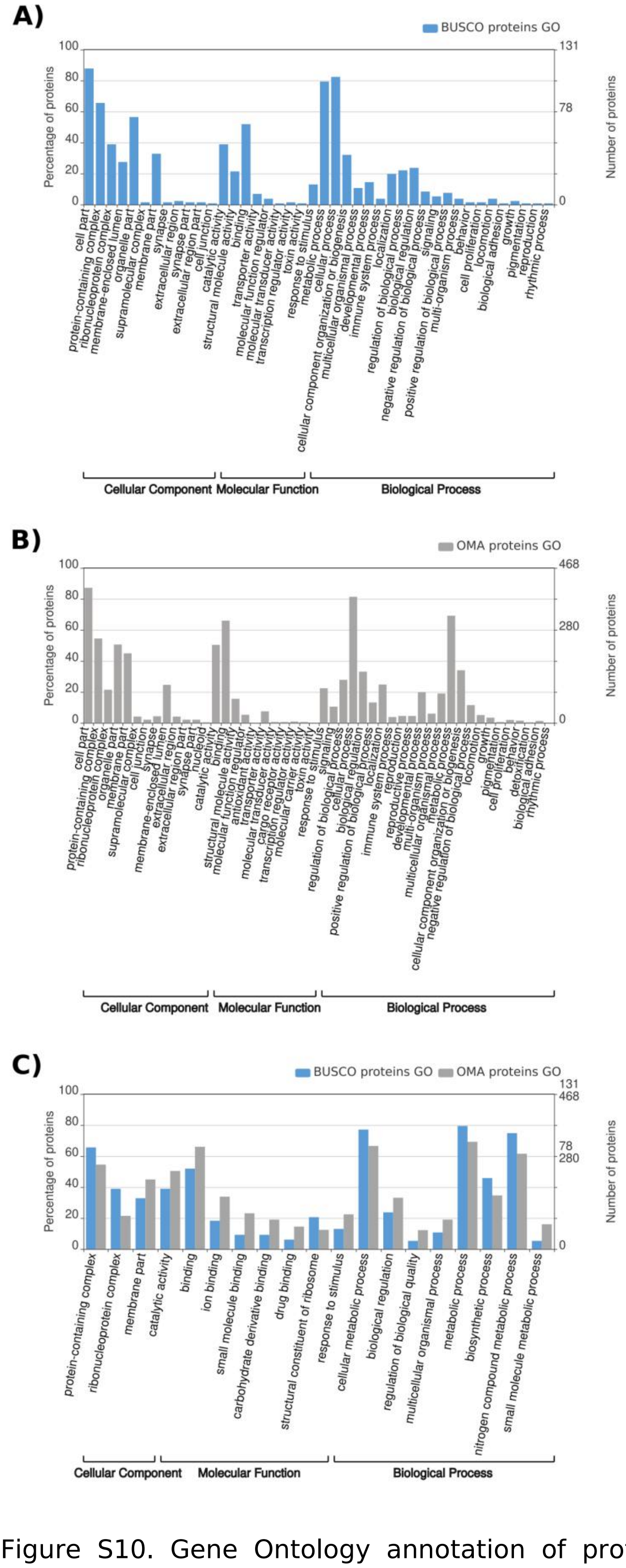

Supplementary Figure S10. Gene Ontology annotation of proteins used in the phylogenomic analysis. A) GO of proteins obtained from BUSCO. B) GO of proteins obtained from OMA. C) GO term enrichment between BUSCO and OMA proteins.

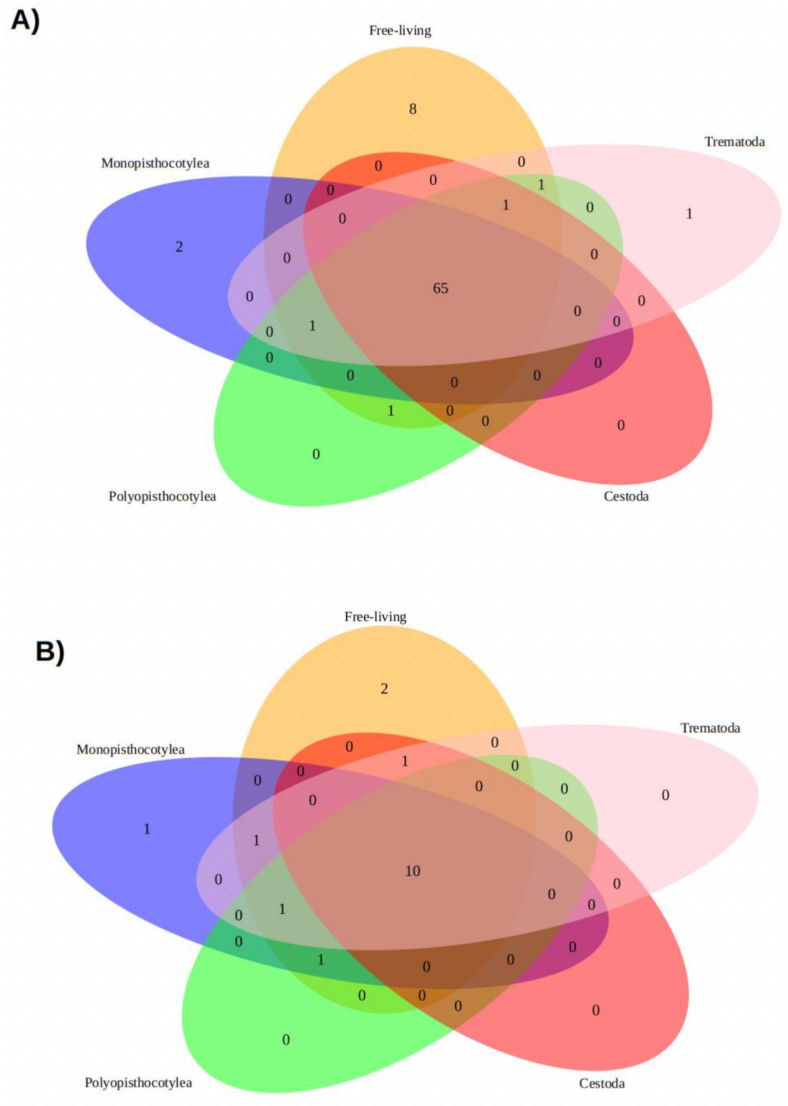

Supplementary Figure S11. Number of shared peptidase families. A) Peptidase families. B) Peptidase inhibitor families in taxonomic groups within the Neodermata.
