# Supplementary material for: Exploring Evolutionary Relationships within Neodermata Using Putative Orthologous Groups of Proteins, with Emphasis on Peptidases
Source: Trop Med Infect Dis. 2023 Jan 12;8(1):59. doi: 10.3390/tropicalmed8010059 (PMC9860727; doi:10.3390/tropicalmed8010059)
Supplement: Supplementary file 1 [file tropicalmed-08-00059-s001.zip › Supplement_R4/Supplementary Results.pdf]

Exploring Evolutionary Relationship within Neodermata using Putative Orthologous Groups of Proteins, with Emphasis on Peptidases

Víctor Caña-Bozada[1], Mark W. Robinson[2], David I. Hernández-Mena[3], Francisco N. Morales-Serna[4]

[1]Centro de Investigación en Alimentación y Desarrollo, A.C., Mazatlán 82112, Sinaloa, Mexico; [2]School of Biological Sciences, Queen's University Belfast, 19 Chlorine Gardens, Belfast, Northern Ireland, UK; [3]Centro de Investigación y de Estudios Avanzados, Instituto Politécnico Nacional, Unidad Mérida, Mérida 97310, Yucatán, Mexico; and [4]Instituto de Ciencias del Mar y Limnología, Universidad Nacional Autónoma de México, Mazatlán 82040, Sinaloa, Mexico

Corresponding author: Francisco N. Morales-Serna,

neptali@ola.icmyl.unam.mx

Supplementary Results

Phylogenetic analysis of BUSCO and OMA OGPs

Annotation of OGPs

The most abundant BUSCO OGPs were Ribosomal protein (27 proteins), Eukaryotic translation initiation factor (4 proteins), and Pre-mRNA-splicing factor (3 proteins) (Supplementary Table S1). The most abundant OMA OGPs were 60S ribosomal protein (26 proteins), 40S ribosomal protein (16 proteins), Eukaryotic translation initiation factor (14 proteins), Proteasome subunit (12 proteins), NADH dehydrogenase (7 proteins), Ras-related protein (7 proteins), Serine/threonine-protein phosphatase (6 proteins), 26S proteasome non-ATPase regulatory subunit (6 proteins), 26S proteasome regulatory subunit (6 proteins), T-complex protein 1 subunit (6 proteins), and V-type proton ATPase subunit (6 proteins) (Supplementary Table S2).

Within BUSCO and OMA OGPs, the most represented GO terms were binding (GO:0005488) and catalytic activity (GO:0003824) within the Molecular Function category; cellular process (GO:0009987) and metabolic process (GO:0008152) within the Biological Process category; and cell part (GO:0044464), protein-containing complex (GO:0032991), organelle part (GO:0044422), ribonucleoprotein complex (GO:1990904), and membrane part (GO:0044425), within the Cellular Component category (Supplementary Figure S10).

Phylogenetic analysis of peptidases

Peptidase orthologous groups

A total of 531 OGs of peptidases and peptidase inhibitors were found in all the platyhelminths (Supplementary File 4). Information about the OGs is shown in Supplementary Table S7. Eighteen OGs were shared among all the species, of which nine OGs were of single-copy. These nine OGs were represented mainly by metallo and threonine peptidases. Two OGs were

shared among parasitic but absent in free-living species (HOG00197, S01; HOG00205, M03).

Peptidases

A total of 3960 peptidases were identified in the 13 species of platyhelminths, distributed in the five major classes (114 aspartic, 1321 cysteine, 1182 metallo, 1057 serine, 258 threonine, and 28 mixed proteins) and 80 families (Supplementary Table S3). For the monogeneans *R. viridisi, S. longicornis, G. salaris, N. melleni, E. nipponicum,* and *P. xenopodis*, the number of peptidases was 159, 265, 289, 186, 293, and 296, respectively. For the cestodes *E. multilocularis, H. microstoma,* and *T. asiatica* were 244, 241, and 293, respectively. For the trematodes, *F. hepatica* and *S. mansoni* were 360 and 280, respectively. For the free-living platyhelminths, *B. semperi* and *S. mediterranea* were 532 to 522, respectively (Supplementary Figure S2A).

Serine peptidases were the most abundant class (Supplementary Figure S3 and Supplementary Table S4) and represented >28% of the total peptidases/peptidase inhibitors in each species of monopisthocotylean monogeneans, whereas in other flatworms represented < 22.3%. Cysteine peptidases were the most abundant class in all the species of polyopisthocotylean monogeneans, whereas for cestodes, trematodes, and free-living platyhelminths cysteine peptidases were the most abundant in some species (such as *E*. *multilocularis*, *H*. *microstoma*, *F*. *hepatica*, *B*. *semperi*, and *S*. *mediterranea*) whilst metallo peptidases were more dominant in others (e.g. *S*. *mansoni* and *T*. *asiatica*).

The most abundant peptidase families in all the platyhelminths studied were C19 (ubiquitinyl hydrolases) with 395 members, S01 (chymotrypsin family) with 324 members, S09 (prolyl oligopeptidases) with 286, C01 (papain family) with 212 members, and T01 (proteasome family) with 194 members (Figure 2 and Supplementary Table S5). In the free-living species, C19 showed a great expansion of proteins (> 62 proteins in *B. semperi* and *S. mediterranea*), in comparison with other platyhelminths (<37 proteins) (Supplementary Table S5). The family S01 was much more represented in monopisthocotyleans (11.78%-30.58%), than in polyopisthocotyleans (4.52%-5.11%), free-living (5.24%-6.21%), trematodes (3.40%-4.29%), and cestodes (2.02%-3.75%) (Supplementary Table S5). Of the 80 families of peptidases, 65 were shared among all the classes of platyhelminths (Supplementary Figure S11A). Free-living species covered 77 of 80 families of peptidases. Eight families of peptidases were specific for free-living species (A02 (retropepsin family), C67, M11 (gametolysin family), M54, M76, M87, S49 (protease IV family), S66), two for monopisthocotyleans (M15, M23), and one for trematodes (M103). The specific peptidases of monopisthocotyleans and trematodes were detected only in a single species.

Peptidase inhibitors

A total of 667 peptidase inhibitors belonging to 17 families were identified in all the platyhelminths (Supplementary Table S3 and Supplementary Table S5). For the monogeneans *R. viridisi, S. longicornis, G. salaris, N. melleni, E. nipponicum,* and *P. xenopodis,* the numbers of peptidase inhibitors were 25, 38, 42, 20, 59, and 36, respectively; for the cestodes *E. multilocularis, H. microstoma*, and *T. asiatica* were 53, 52, and 56; for the trematodes *F.*

hepatica and *S. mansoni* were 52 and 46; for the free-living species *B. semperi* and *S. mediterranea* were 80 and 108 (Supplementary Figure S2B). The peptidase inhibitors were most highly represented in cestodes (>16%) than in the other platyhelminths (<14%), except for *S. mediterranea* (17.1%) (Supplementary Figure S3).

The I02 (kunitz) family was the most abundant with 173 members (Supplementary Figure S3 and Supplementary Table S5). This family was more represented in cestodes (5.44%-7.07%) than in the other platyhelminths (<3.99%), except for *E. nipponicum* (6.53%). Of the 17 families of peptidase inhibitors, 10 were shared among all the species of platyhelminths (Supplementary Figure S11B). Two families were specific for free-living species (I17 (WAP-type family), I31 (thyropin family)), and one for monopisthocotyleans (I15 (antistasin family)). The peptidase inhibitors of free-living species covered 16 of the 17 families.

Secreted peptidase and peptidase inhibitors

SignalP predicted 565 peptidases (14.27%) and 237 peptidase inhibitors (35.53%) as classically secreted proteins (Supplementary Table S6). In each platyhelminth species, the peptidase inhibitors were more represented by proteins with signal peptide than the peptidases (Figure 3). The I02, I93, C01, M12, and S01 families presented a high proportion of protein with signal peptide in comparison with other families (> 40% of proteins with signal peptide) (Supplementary Figure S4). The proportion of proteins with signal peptide was similar between parasites and free-living. In addition, 79 peptidases (2%) and 18 peptidase inhibitors (2.7%) were predicted as non-classical secreted proteins.
